# A VOSviewer-Based Bibliometric Analysis of Prescription Refills

**DOI:** 10.3389/fmed.2022.856420

**Published:** 2022-06-21

**Authors:** Runchen Fu, Haiping Xu, Yongjie Lai, Xinying Sun, Zhu Zhu, Hengchang Zang, Yibo Wu

**Affiliations:** ^1^School of Pharmaceutical Sciences, Shandong First Medical University, Tai’an, China; ^2^School of Pharmaceutical Sciences, Shandong University, Jinan, China; ^3^School of Public Health, Peking University, Beijing, China; ^4^Pharmaceutical Preparation Section, Chinese Academy of Medical Sciences and Peking Union Medical College, Beijing, China; ^5^NMPA Key Laboratory for Technology Research and Evaluation of Drug Products, School of Pharmaceutical Sciences, Shandong University, Jinan, China

**Keywords:** prescription refills, research trend, VOSviewer, web of science core collection, bibliometric analysis

## Abstract

**Purpose:**

Prescription refills are long-term prescriptions for chronic patients in stable status, which varies from country to country. A well-established prescription refill system is beneficial for chronic patients’ medication management and facilitates the efficacy of clinical care. Therefore, we carried out a bibliometric analysis to examine the development of this field.

**Summary:**

Publications on prescription refills from 1970 to 2021 were collected in the Web of Science Core Collection (WoSCC). Search strategy TS = “prescri* refill*” OR “medi* refill*” OR “repeat prescri*” OR “repeat dispens*” OR TI = refill* was used for search. VOSviewer was applied to visualize the bibliometric analysis. A total of 319 publications were found in WoSCC. Study attention on prescription refills has shown a steady rise but is still low in recent years. The United States was the most productive country, which had the highest total citations, average citations per publication, and the highest H-index, and participated in international collaboration most frequently. The University of California system was the most productive institution. The U.S. Department of Veterans Affairs was the institution with the most citations, most average citation, and highest H-index. Sundell was the most productive author, and Steiner J. F. was the most influential author. “Adherence,” “medication,” and “therapy” were the most prominent keywords.

**Conclusion:**

Publications on prescription refills have increased rapidly and continue to grow. The United States had the leading position in the area. It is recommended to pay closer attention to the latest hotspots, such as “Opioids,” “Surgery,” “Differentiated care,” and “HIV.”

## Introduction

A prescription refill is a long-term prescription for chronic patients in stable status. In the United States and Europe, prescription refill policy has been a general tool in the national health insurance system with specific laws and policies (1). Prescription refill in the United States is prescribed by physicians and reviewed by pharmacists. Hospitals and pharmacies in different states have different prescription refill categories responding to the Code of Federal Regulations (CFR) (2). In the United Kingdom, prescription refill, also called repeat prescription, is prescribed by general physicians and reviewed by pharmacists. However, the latter with recognized qualifications can become an independent prescriber. Unlike in the United States, the United Kingdom does not have definite prescription categories and only defines some drugs which cannot be prescribed with prescription refills. It created repeat prescribing risk assessment tools used for evaluating patients’ conditions and guaranteeing medication safety (3). Moreover, some countries like Australia and Singapore also have well-established prescription refill services (4, 5).

However prescription refills in some developing countries were still in the exploration phase, and the system of prescription refills has not been established yet, for example, in China. Since 2015, some regions in China have begun to provide the prescription refill service, but different regions have different regulations on expiration dates, types of disease, and drugs in the prescription refill system (1). On 10 August 2021, China promulgated the Long-Term Prescription Management Specification (Trial) to define the applicable population of prescription refills, the prescriber, the prescription refill process, etc. It standardizes the prescription refill system (6). However, compared with European countries and the United States, it is still faced with challenges. The prescription refills are only prescribed by general practitioners. Furthermore, the scope of drugs and diseases needs to be extended.

Bibliometrics, proposed by Prichard, is employed to review the literature and predict the development of scientific research, by applying literature systems and literature metrology characteristics as research objects, and using statistical methods to study quantity relationships and laws between literature and literature systems (7). It can present the trend of an area and the most influential research results rapidly and accurately, establishing a theoretical basis for further study.

Based on a bibliometric analysis conducted on the Web of Science Core Collection (WoSCC), the study analyzed various literature on prescription refills published between 1970 and 2021 using the VOSviewer from the perspective of co-occurrence and clustering. We aimed at providing the research status, trend, and forefront in the study of prescription refills to provide the reference for the researchers’ follow-up research.

## Methods

### Data Source and Retrieval Strategy

We performed a bibliometric analysis in WoSCC, a database strictly evaluating publications and updating them adequately to provide the most influential and reliable information (8, 9).

The retrieval strategy was as follows: TS = “prescri* refill*” OR “medi* refill*” OR “repeat prescri*” OR “repeat dispens*” OR TI = refill*.

### Screening Criteria

**FIGURE 1:**
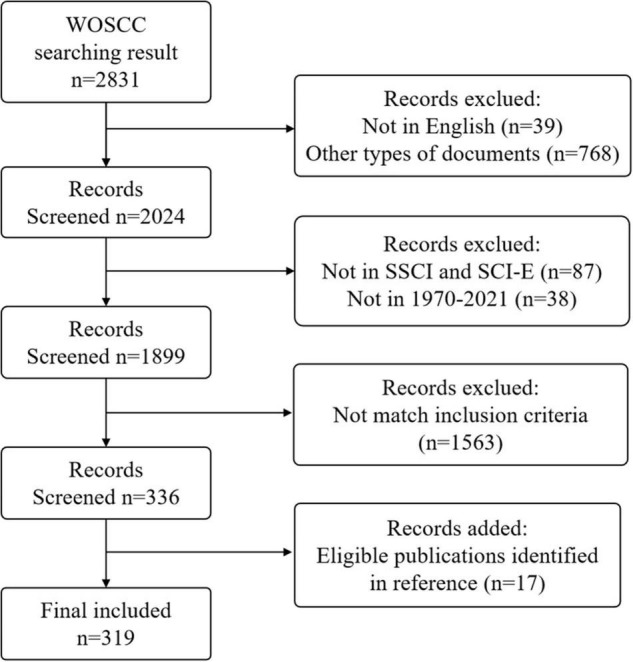
Flowchart of included and excluded publications. Other types of documents included meeting abstracts, proceedings papers, letters, review articles, editorial materials, notes, new items, corrections, early access, book reviews, and book chapters.

The screening flowchart is shown in Figure 1. It was found that several manuscripts were published before 1970 in pre-search. Considering problems in report forms and a lack of information, the search starting time was set as 1970.

The articles were retrieved and screened by two researchers, and the criteria for screening and inclusion were: (1) language was “English,” (2) publication type was “article,” (3) data source was WoSCC (SCI-E, SSCI), (4) published from 1 January 1970 to 31 December 2021, (5) the topic was prescription refills. Additionally, references of all included studies were searched.

To make sure the topic of the included literature was prescription refills, two researchers, based on back-to-back methods, read abstracts of the retrieved articles and excluded 520 manuscripts that were unrelated to medicine, such as materials science (101), engineering (101), metallurgy (52), chemistry (46), physics (41), environmental sciences (30), and plant sciences (27). The two researchers read the full text of articles in the field of medicine and excluded about 230 articles whose objects or aim of research were not prescription refills.

In the end, 319 manuscripts were included, and the detailed information of the manuscripts included was presented in Supplementary Material 1.

### Data Preparation and Information

The final literature was exported for analysis. Indicators for analysis included the number of publications, average citations per publication, countries, institutions, journals, keywords, authors, and the H-index (h papers published in the journal have been cited at least h times), among which the number of publications, average citations per publication, and the H-index were obtained from the Citation Report in the Web of Science. Bradford’s law was used to identify and analyze core journals. In Bradford’s law, if journals were ranked in decreasing order of a number of publications in a specific discipline, then journals were classified into “core journals,” “related journals,” and “non-related journals” groups, respectively, with the same amount of publications and each group has the number of journals as 1:n:n^2^ (10).

### Statistical Analysis

Microsoft Excel 2016 were used to analyze and draw the number of publications, the average number of citations per publication, and the H-index. Meanwhile, Microsoft PowerPoint 2016 was applied to draw the flowchart of literature screening. Literature was imported to the VOSviewer (version 1.6.16, Leiden University Center for Science and Technology Studies, Leiden, Netherlands) to draw co-occurrence maps of countries, authors, and keywords.

The statistical package SPSS (IBM SPSS 21.0, SPSS Inc., Chicago, IL, United States) was used to analyze the trend of publications using a logistic growth model *f*(*x*) = *a*/[1 + *b* × exp(*^–^c* × *x*)] (11), which has better fitting accuracy for literature in a specific field (12). The time trend was described by year. A logistic regression model *f*(*x*) = *a*/[1 + *b* × exp(*^–^c* × *x*)] was used to fit the cumulative number of publications, with the point of maximum growth being the inflection point of the curve *T* = ln(*b*/*c*) (11), and *x* representing a specific year and *f*(*x*) representing the cumulative number of publications for this year.

## Results

### Publications

**FIGURE 2:**
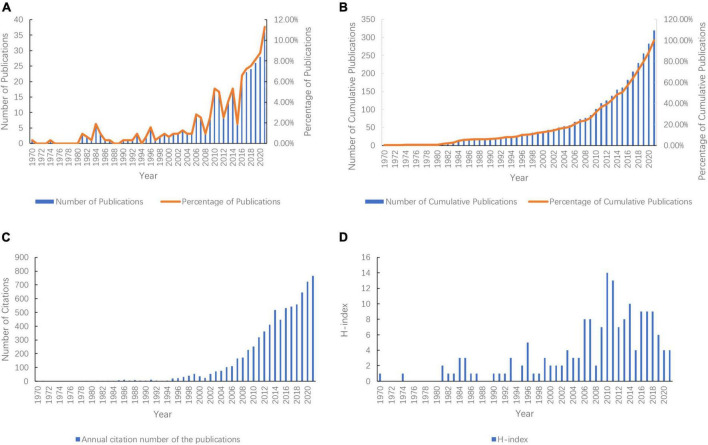
Global number of publications, number of citations, and H-Index of publications in the field of prescription refills from 1970 to 2021. **(A)** Annual number of publications and their percentage; **(B)** Number and percentage of the annual cumulative number of publications; **(C)** Annual citation number of the publications; **(D)** Annual H-Index of the publications.

This study included 319 publications published from 1970 to 2021 in WoSCC. Trends in the number of publications are shown in Figure 2A. The annual cumulative number of publications trend (Figure 2B) roughly fits the logistic model {*f*(*x*) = 934.268/[1 + 554.375exp (−0.107*x*)] (*R*^2^ = 0.981)}. It can be predicted from the model that the growth rate of publications might be the highest in 2028 [*T* = ln(*b*/*c*) = ln55(4.375/0.107) = 59.04] and gradually decline after 2028 with a continual increase in the cumulative number of publications.

By 31 December 2021, the publications included had been cited 7,349 times, with an average of 23.41. The annual citation number of the publications has stayed at a high level (more than 300 times) for a decade and continued to rise (Figure 2C). The H-index of publications included was 170, with the two highest H-index in 2010 and 2011 (Figure 2D).

### Countries

**FIGURE 3:**
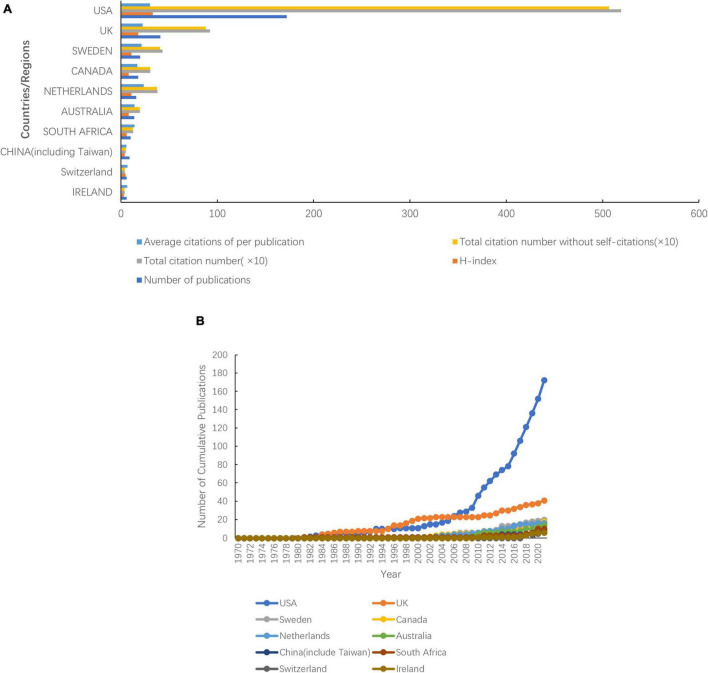
Top 10 productive countries from 1970 to 2021. **(A)** Total number of publications, citations, citations without self-citations, average citations per publication, and H-index. **(B)** Number of cumulative publications in various countries.

**FIGURE 4:**
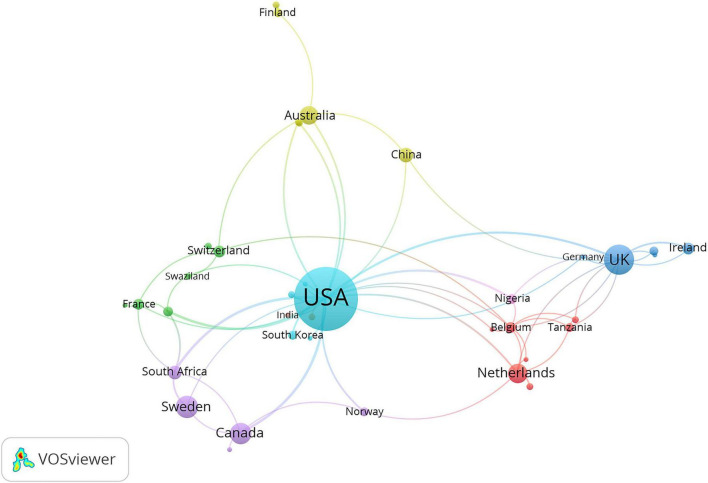
International collaboration between countries. The countries were labeled using different colors and the links represented international collaborations.

A total of 47 countries or regions published articles related to prescription refills. The United States was the most productive country with 172 publications, followed by the United Kingdom (41 publications), Sweden (20 publications), Canada (18 publications), the Netherlands (16 publications), Australia (14 publications), South Africa (10 publications), China (9 publications), Ireland (6 publications), and Switzerland (6 publications) (Figure 3B). The United States (33), the United Kingdom (18), Sweden (11), and the Netherlands (11) ranked as the top three high H-index countries, while the United States was top 1 (Figure 3A). The top three countries in the total number of citations were the United States (5,191 citations and 5,067 without self-citations), the United Kingdom (923 citations and 882 without self-citations), and Sweden (432 citations and 406 without self-citations), whereas the United States (30.18 citations), the Netherlands (23.81 citations), and the United Kingdom (22.84 citations) were the top three countries in average citations per publication (Figure 3A). The United States participated in international collaboration most frequently, followed by the United Kingdom and Sweden (Figure 4).

### Institutions, Authors, and Journals

**FIGURE 5:**
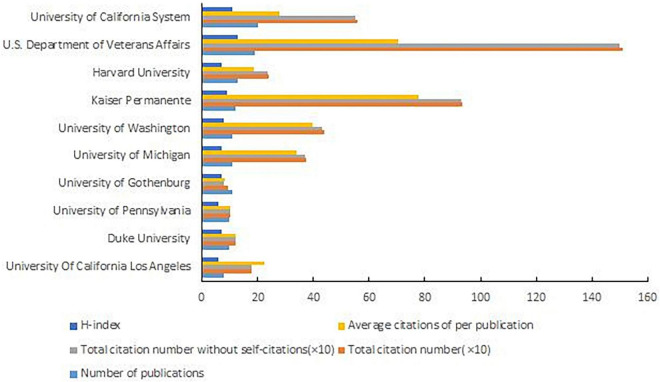
Number of publications, total citation number, total citation number without self-citations, average citations per publication, and H-Index of the Top 10 reproductive institutions.

The University of California system was the most productive institution with 20 publications, followed by the U.S. Department of Veterans Affairs (19 publications), Harvard University (13 publications), and others (Figure 5). The U.S. Department of Veterans Affairs was the institution with the most citations (1,499 citations) and Kaiser Permanente was the most average citations per publication (77.83 citations) (Figure 5). The top three institutions in the H-index were the U.S. Department of Veterans Affairs, the University of California system, and Kaiser Permanente (Figure 5).

**FIGURE 6:**
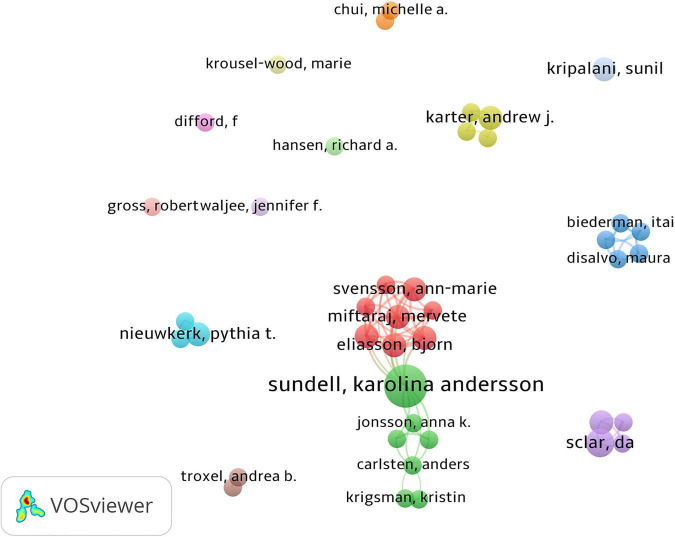
Co-authorship among authors. Dots represented authors, larger dot indicated a higher number of publications, the clusters were labeled using different colors, and the links represented author collaborations.

The top three productive authors are shown in Table 1. We only listed the top three authors, because there were many authors tied for the fourth place with four publications. Sundell (nine publications) was the most productive author. Steiner published the most influential article with 939 citations, with only one publication about prescription refills. A co-authorship map was generated as shown in Figure 6, which included authors publishing more than three articles. The intensive clusters in the map indicated that cooperation between authors was close and they formed many research teams.

**TABLE 1:** Publication number, total citation number, total citations without self-citations, average citations per publication, and H-Index of the top three reproductive authors.

Authors	Records	Total citations	Total citations without self-citations	Average citations per publication	H-index
Sundell	9	89	76	9.89	7
Nilsson	6	224	215	37.33	6
Sclar	5	239	239	47.8	5

The 319 articles were published in 148 journals. Based on Bradford’s law, nine journals were defined as “core journals” in the area (Table 2). The most productive journal was the Journal of the American Pharmacists Association (14 publications). The British Medical Journal ranked top 1 in total citations and average citations per publication, respectively.

**TABLE 2:** Categories, publication number, total citations, total citations without self-citations, average citations per publication, and H-Index of the top nine reproductive journals.

Journals	Categories	Records	Total citations	Total citations without self-citations	Average citations per publication	H-Index
Journal of the American Pharmacists Association	Pharmacology and pharmacy	14	130	125	9.29	6
Pharmacoepidemiology and Drug Safety.	Public, environmental and occupational health pharmacology and pharmacy	12	303	298	25.25	8
British Medical Journal	Medicine, general and internal	9	312	310	34.67	6
British Journal of General Practice	Primary health care medicine general and internal	9	199	194	22.11	5
Journal of Managed Care and Specialty Pharmacy	Pharmacology and pharmacy health care sciences and services	7	38	38	5.43	3
Journal of the International AIDS Society	Infectious diseases immunology	8	117	115	14.63	5
Patient Preference and Adherence	Medicine, general and internal	7	87	86	12.43	5
Journal of Clinical Pharmacy and Therapeutics	Pharmacology and pharmacy	6	207	207	34.83	6
Plos One	Multidisciplinary sciences	6	75	75	12.5	3

### Highly Cited Articles

The top five cited articles are shown in Table 3: *The assessment of refill compliance using pharmacy records: methods, validity, and applications; measurement of adherence in pharmacy administrative databases: a proposal for standard definitions and preferred measures; factors associated with medication refill adherence in cardiovascular-related diseases: a focus on health literacy; adherence in glaucoma: objective measurements of once-daily and adjunctive medication use; medications scale (ARMS) among low-literacy patients with chronic disease.*

**TABLE 3:** The top 5 most cited publications of prescription refill.

Title	Author (lead author)	Journal	Citation	Average citation
The assessment of refill compliance using pharmacy records: Methods, validity, and applications	Steiner	Journal of Clinical Epidemiology	939	36.12
Measurement of adherence in pharmacy administrative databases: A proposal for standard definitions and preferred measures	Hess and Lisa	Annals of Pharmacotherapy	522	30.71
Factors associated with medication refill adherence in cardiovascular-related diseases: A focus on health literacy	Gazmarariana and Julie	Journal of General Internal Medicine	249	14.65
Adherence in glaucoma: Objective measurements of once-daily and adjunctive medication use	Robin and Alan	American Journal of Ophthalmology	236	14.75
Development and Evaluation of the Adherence to Refills and Medications Scale (ARMS) among Low-Literacy Patients with Chronic Disease	Kripalani and Sunil	Value in Health	176	12.57

### Keywords

Keywords can be analyzed to find research hotspots and core content. Keywords such as “medication” were combined or unified to obtain a better perspective. For example, “medication adherence,” “persistence,” “compliance,” and “patient adherence” were unified as “adherence.” VOSviewer was used to visualize the frequency of keywords. A total of 78 keywords whose frequency was at least five times were analyzed.

**FIGURE 7:**
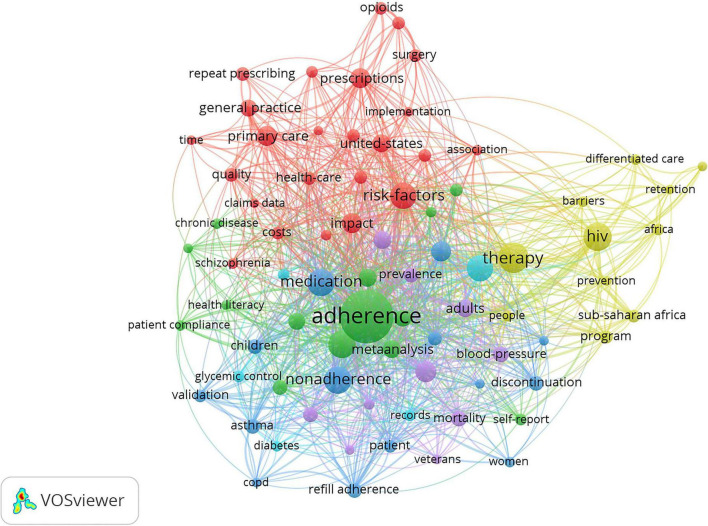
Network visualization of the keywords.

**FIGURE 8:**
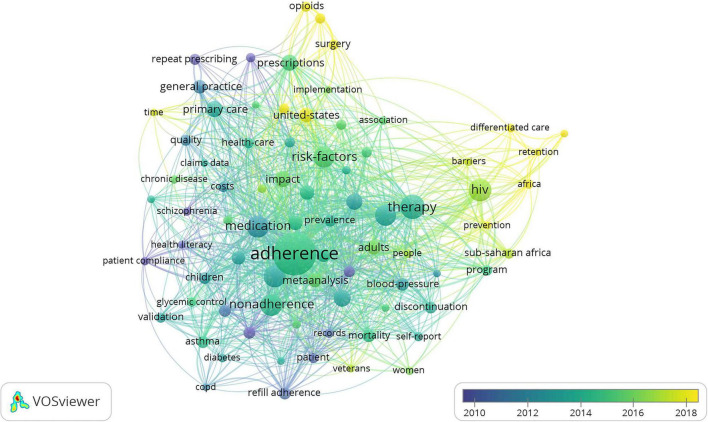
Time-based visualization of keywords variation.

“Adherence,” “medication,” and “therapy” were the most prominent keywords (Figure 7), indicating almost all studies revolved around these three keywords. Keywords included can be classified into six clusters as follows: (1) primary healthcare systems on prescription refills, (2) study on the compliance of patients with chronic disease, (3) prescription refills of patients with asthma, (4) prescription refills of patients with AIDS, (5) prescription refills of patients with hypertention, and (6) prescription refills of patients with diabetes. As the keywords of some clustering nodes in Figure 7 were not completely displayed, we listed the important keywords in items generated by VOSviewer, as shown in Table 4. Time-based visualization of keyword variation was presented by VOSviewer according to the development of keywords over time (Figure 8). Keywords in purple appeared earliest and keywords in yellow appeared latest. The keywords “Opioids,” “Surgery,” “Differentiated care,” “HIV,” “Barriers,” and “Africa” are represented by their most recent appearance. We can know the research edge of prescription refills through the updated keywords as well.

**TABLE 4:** Keywords clusters in the field of prescription refill.

Items	Keywords
1.	General practice; health-care; prescriptions; primary care; risk-factors; and united-states
2.	Adherence; chronic disease; community pharmacy; drug-therapy; and patient compliance
3.	Asthma; discontinuation; management; non-adherence; and refill adherence
4.	Africa; differentiated care; HIV; prevention; and therapy
5.	Blood-pressure; hypertension; interventions; mortality; and pharmacy records
6.	Care; diabetes; disparities; glycemic control; and records

## Discussion

Based on the bibliometrics and the visualization software, we aimed to explore the research trends and hotspots in the field of prescription refills from 1970 to 2021. The total number of publications from 1970 to 1994 was 25, and the average annual number of publications was 1, indicating very low attention on prescription refills in the 25 years. As this period was the beginning of literature research, growth of the literature was slow. From 1995 to 2009, the total number of publications was 59, and the average annual number of publications was 3.9 with a slow upward trend. After 2010, publications began to significantly increase and exceeded 20 publications in 2016. It can be seen that study attention on prescription refills has shown a steady rise. The number of publications showed a growth trend, and 2028 might be the year with the highest publication growth rate. This research field may remain a hotspot in the next few years. According to the analysis of the H-index, we found that the highest H-index was 14 in 2010, and the number of publications in that year was 17, indicating that the publications of this year were of great value to the field of prescription refills and deserved more attention. The highest number of citations in 2021 demonstrated an increasing emphasis on prescription refills. The H-index had decreased as the data in the last 5 years were close to the data collection time (31 December 2021). However, the publications in recent years would have more citations.

The publications not only presented a dynamic time trend varying with the years but also showed varieties among different countries. The United States, the United Kingdom, and Sweden ranked as the top three productive countries, accounting for 73.04% of the total publications. The United States showed its dominant position in this research field with excellent performance in publication outputs, H-index, total citations, and average citations per publication. As a country with most international cooperation, the United States has cooperation projects with African countries, especially in HIV research. It conducted a study with Botswana on the impact of pharmacy supplement data on the outcome of virology treatment for HIV-infected adolescents in Botswana (13). The United States also had joint projects related to AIDS research with Kenya and Zimbabwe (14, 15).

The prescription refill system is closely related to the development of hospital pharmacy. Clinical pharmacy originated in the United States in the 1960s and was introduced in the United Kingdom in the early 1970s (16), which was a breakthrough in the development of hospital pharmacy. Since then, pharmacists have had the capacity for prescribing, medication management, patient education, etc. These countries with developed hospital pharmacies have a well-established prescription refill system. However, hospital pharmacy in China and South Africa started in the 1980s and their development was limited by the level of the medical system, leading to the undeveloped prescription refill system. (17, 18). In addition, due to the limitations of the included article language, few studies from Japan and other countries were included. In fact, Japan has a mature repeat prescription system.

Europe, the United States, and other countries have established a mature prescription refill system, which has become a basic tool of the national health insurance system, with specific laws and regulations to improve the implementation of policies. In the United States, prescription refills were prescribed by doctors for patients with chronic diseases in stable status and in need of long-term drug treatment. Pharmacists reviewed prescription refills, recorded, and followed up on patients’ medication. Hospitals and pharmacies throughout the country have prescribed categories of drugs for long-term use. Prescription refills were valid for 12 months. In addition, the details of refill management were mandated in Section 22 of Part 1306 of the Code of Federal Regulations (2). In the United Kingdom, prescription refills are prescribed by general practitioners and reviewed by pharmacists, but pharmacists also can be upgraded to independent prescribers after training and examination. There were no clear lists of prescriptions, and only some drugs that could not be used for long-term prescriptions were stipulated. The prescription duration was at most 12 months. In addition, the United Kingdom also had a risk assessment tool to assess the patient’s condition and ensure the safe use of drugs (3).

However, in some developing countries, prescription refills are still in the exploration stage without a complete prescription refill system. For example, China has carried out a pilot policy in several cities since 2015, but it was confronted with the following issues: prescription refills were only prescribed by general practitioners; few diseases and medicines were included; durations of prescriptions were short; and there was no corresponding legislation (1). It needs further exploration. Therefore, the mature systems of European countries and the United States are good references. China has just issued the trial version of the policies, but only general practitioners issued it. It can refer to the developed systems to strengthen the training and construction of pharmacists, increase the role of pharmacists in the service of prescription refills, reduce the burden of doctors, and promote cooperation between doctors and pharmacists.

Among the top 10 academic institutions, except the University of Gothenburg, the other nine institutions were all located in the United States, indicating that the U.S. academic institutions had high productivity in this field. The most productive institution was the University of California, which focused on HIV antiretroviral therapy adherence (19, 20). Besides, the research team also centered on drug replenishment systems, such as automatic dispensing system. They found the wholesaler-to-ADC direct refill program, which included prepackage and bar-code-assisted supplements, decreased ADC refill errors (21). Research on online drug supplementation systems was also the focus of the institution research (22, 24). As the second most productive institution, the U.S. Department of Veterans Affairs, has well-established mail-order pharmacy systems and has done more research on services such as pharmacy intervention (23, 25, 26). It was worth knowing that Caesars Healthcare was a commercial operating organization whose research focuses on text messages, phone reminders for medication replenishment, and online drug supplements (24, 27, 28).

Sundell, Jonsson, Lesen, and Mardby have formed a group of authors with close cooperation, and they have been leaders in the research field. Their collaborative research focused on the comparison of different drug supplementation methods and the effects of drug substitution on the effectiveness of prescription refills (29–31). Meanwhile, according to the number of publications, citations, H-index, and other indicators, the Journal of the American Pharmacists Association, Pharmacoepidemiology and Drug Safety, British Medical Journal, and other journals were recognized as popular journals in the field of prescription refills. Most articles on prescription refill were published in Pharmacology and Pharmacy, Medicine General Internal, Public Environment Occupational Health, Health Care Sciences Services, and Primary Health Care.

In influential institutions, researchers in the field can seek cooperation and enhance research exchanges. In addition, researchers can explore novel ideas by focusing on the research directions of influential authors. Influential journals in prescription refills can draw researchers from many countries to know the trends in this field and communicate with each other on these platforms.

*The assessment of refill compliance using pharmacy records: Methods, validity, and applications*, which is the most cited article, was written by Steiner. It was a review of the pharmacy record database, whose results showed significant associations between refill compliance and other adherence measures, as well as measures of drug presence (e.g., serum level of drugs) or physiological drug effects (32). The second most cited article, *Measurement of adherence in pharmacy administrative databases: A proposal for standard definitions and preferred measures*, compared five compliance evaluation methods, and suggested that Medication Refill Adherence was the preferred method of adherence using administrative data (33). The research, whose title was *Factors associated with medication refill adherence in cardiovascular-related diseases: A focus on health literacy*, showed that race/ethnicity, education, and program complexity were all associated with medication supplement compliance (34).

Keywords can help researchers understand the frontier trends and grasp the research direction. Analysis of the collinear relationship among keywords can classify the keywords into the following six major clusters: (1) primary healthcare systems on prescription refills, (2) studies on the compliance of patients with chronic disease, (3) patients with hypertention with asthma, (4) prescription refills of patients with AIDS, (5) prescription refills of patients with hypertension; (6) prescription refills of patients with diabetes. These six clusters will be the main focus of prescription refills in the future. Asthma, AIDS, hypertension, and diabetes were the top four types of diseases in prescription refill research. Studies have shown that interventions such as pharmacist-led interventions, regular follow-up, text message alerts, and scheduled drug programs at community pharmacies contributed to increased drug supplementation rates for these four diseases (35–39). In addition, by reading the relevant literature, it was found that the pharmacy record database was a good reflection of the supply and demand of drugs, as well as the prescription refills for patients. The database of pharmacy records allows pharmacists to determine the pattern of drug distribution and the durability of treatment over time (40).

The research methods included in the study can be roughly divided into three categories: first, the analysis of patients’ adherence to the advantage side through the pharmacy record database (40, 41); second, questionnaires, interviews, patients’ self-reports, and other survey methods were used to analysis (42, 43); third, analysis was performed based on the patient’s treatment compliance data (40–44). A study about differentiated service delivery for HIV treatment in South Africa found that antiretroviral treatment adherence clubs facilitated medication adherence to reinforce counseling and track patients who did not come for a follow-up. However, both actions were faced with challenges (45). A cross-sectional study in Northern California that examined the relationship between medication adherence and doctor-patient communication among 9,377 patients with diabetes using self-reports, indicated poor communication ratings were independently associated with objectively measured inadequate cardiometabolic medication refill adherence, particularly for oral hypoglycemic medications (46). In addition, Duru et al. found that patients who received medication refills by mail were more likely to have good adherence than patients who obtained refills at offline pharmacies in antiglycemic, antihypertensive, or lipid-lowering medications (23). Based on data from the Centers for Medicare and Medicaid Services, Vaidya et al. discovered that adherence of patients with asthma may be related to race/ethnicity, combined diseases, and the type of Medicaid program (47).

At the same time, we analyzed the frontiers and hotspots of prescription refills. Results showed that the keywords “Opioids,” “Surgery,” “Differentiated care,” “HIV,” and “Barriers,” “Africa” have emerged in the last 5 years. It can be seen that the differentiated care of AIDS and the application of postoperative opioids have become a hotspot in the field of prescription refills. The study by Solouki et al. discussed the application of opioids in postoperative analgesia (48). The study by Lee et al. discussed the impact of education and prescribing guidelines on reducing postoperative use of opioids (49). AIDS research may have certain regional characteristics. According to the incidence rate, we can know that it was a significant study in African countries, researchers need to actively explore relevant antiretroviral therapy and its influencing factors in the future. The postoperative application of opioids will be a popular research topic as well, and researchers can pay more attention to the standardized application of opioids and other issues. Certainly, this just broadly indicates the general condition of the developmental process and hotspots at present due to a single source of literature, as prescription refills vary widely among countries and regions.

Compared with the traditional method of reading numerous studies and summarizing it to obtain the research status of prescription refills, our bibliometric analysis can provide researchers with an intuitive and quick way to obtain information in this field. Researchers can obtain the required information purposefully according to the contents displayed in the article, which improves the efficiency of the scientific research.

Our research also had some limitations. First, these publications were only derived from the SCI-E and SSCI of the WoSCC database, which might cause relatively scarce retrievals, although the WoSCC database strictly evaluates the literature and is most frequently used for literature metrology analysis (9). Second, we only analyzed the English literature, but retrievals were supplemented by references cited in publications included. Third, although the initial search for literature was reviewed and screened by two researchers, it cannot be ruled out that there was a certain bias in the selection of the literature. We have formulated a series of strict screening principles, therefore, many documents that did not meet the requirements were excluded. Fourth, by using the method of bibliometrics, this study macroscopically analyzed the influence of countries, institutions, journals, authors, and keywords on prescription refills, and revealed the future research trends and hotspots in this field to a certain extent. However, the impact of relevant policies, healthcare systems, and government agencies on the development of prescription refill systems needs to be investigated further.

## Conclusion

Recently, the number of publications on prescription refills has been increasing rapidly and continues to grow. The United States has the leading position in the area. It is recommended to pay closer attention to the latest hotspots, such as “Opioids,” “Surgery,” “Differentiated care,” and “HIV.” These results provide researchers with a visual and quick way to get information about prescription refills.

## Author Contributions

YW and ZZ put forward the idea. RF and YL designed the study. HX and RF collected the literature and wrote the manuscript. YW and HZ revised the manuscript. XS and ZZ prepared the figures and tables. YW and HZ supervised and administrate the project. YW acquired the funding. All authors contributed to data analysis, drafting, and critically revised the study, agreed to be accountable for all aspects of the work, read, and approved the final manuscript.

## Conflict of Interest

The authors declare that the research was conducted in the absence of any commercial or financial relationships that could be construed as a potential conflict of interest.

## Publisher’s Note

All claims expressed in this article are solely those of the authors and do not necessarily represent those of their affiliated organizations, or those of the publisher, the editors and the reviewers. Any product that may be evaluated in this article, or claim that may be made by its manufacturer, is not guaranteed or endorsed by the publisher.
