# Supplementary material for: A VOSviewer-Based Bibliometric Analysis of Prescription Refills
Source: Front Med (Lausanne). 2022 Jun 21;9:856420. doi: 10.3389/fmed.2022.856420 (PMC9254907; doi:10.3389/fmed.2022.856420)
Supplement: Supplementary file 1 [file Data_Sheet_1.docx]

Supplementary Materials

Supplementary Materials 1

1. Alghadeer S, Althunayan SF, Alghamdi BM, Bintaleb D, Alnaim L. Evaluation and pharmacists perspective of repeat prescribing process in refill clinics. Saudi Pharm J. 2021;29(11):1336-42.

2. Begum M, Gonzalez-Chica D, Bernardo C, Woods A, Stocks N. Trends in the prescription of drugs used for insomnia: an open-cohort study in Australian general practice, 2011-2018. Br J Gen Pract. 2021;71(712):E877-E86.

3. Hou CH, Pu C. Medication Adherence in Patients With Glaucoma and Disability. JAMA Ophthalmol. 2021;139(12):1292-8.

4. Amstutz A, Lejone TI, Khesa L, Kopo M, Kao M, Muhairwe J, et al. Offering ART refill through community health workers versus clinic-based follow-up after home-based same-day ART initiation in rural Lesotho: The VIBRA cluster-randomized clinical trial. PLos Med. 2021;18(10):20.

5. McGinnis KA, Skanderson M, Justice AC, Akgun KM, Tate JP, King JT, et al. HIV care using differentiated service delivery during the COVID-19 pandemic: a nationwide cohort study in the US Department of Veterans Affairs. J Int AIDS Soc. 2021;24:7.

6. Phillips TR, Fairley CK, Donovan B, Ong JJ, McNulty A, Marshall L, et al. Sexual health service adaptations to the coronavirus disease 2019 (COVID-19) pandemic in Australia: a nationwide online survey. Aust N Z Publ Health. 2021;45(6):622-7.

7. Brizido C, Ferreira AM, Lopes P, Strong C, Mendes GS, Gama FF, et al. Medication adherence to direct anticoagulants in patients with non-valvular atrial fibrillation - a real world analysis. Rev Port Cardiol. 2021;40(9):669-75.

8. Khawagi WY, Steinke D, Carr MJ, Wright AK, Ashcroft DM, Avery A, et al. Evaluating the safety of mental health-related prescribing in UK primary care: a cross-sectional study using the Clinical Practice Research Datalink (CPRD). BMJ Qual Saf.15.

9. Goyal A, Payne S, Sangaralingham LR, Jeffery MM, Naessens JM, Gazelka HM, et al. Variations in Postoperative Opioid Prescription Practices and Impact on Refill Prescriptions Following Lumbar Spine Surgery. World Neurosurg. 2021;153:E112-E30.

10. Goshtasbi K, Abouzari M, Abiri A, Ziai K, Lehrich BM, Risbud A, et al. Trends and patterns of neurotology drug prescriptions on a nationwide insurance database. Laryngoscope Investig Otol. 2021;6(5):1096-103.

11. Wetzel M, Hockenberry JM, Raval MV. Opioid Fills in Children Undergoing Surgery From 2011 to 2014 A Retrospective Analysis of Relationships Among Age, Initial Days Supplied, and Refills. Ann Surg. 2021;274(2):E174-E80.

12. Ellis JL, Ghiraldi EM, Cohn JA, Nitti M, Friedlander JI, Ginzburg S, et al. Prescribing Trends in Post-operative Pain Management After Urologic Surgery: A Quality Care Investigation for Healthcare Providers. Urology. 2021;153:156-62.

13. Hero C, Karlsson SA, Franzen S, Svensson AM, Miftaraj M, Gudbjornsdottir S, et al. Impact of Socioeconomic Factors and Gender on Refill Adherence and Persistence to Lipid-Lowering Therapy in Type 1 Diabetes. Diabetes Ther. 2021;12(9):2371-86.

14. Chang TJ, Bridges JFP, Bynum M, Jackson JW, Joseph JJ, Fischer MA, et al. Association Between Patient-Clinician Relationships and Adherence to Antihypertensive Medications Among Black Adults: An Observational Study Design. J Am Heart Assoc. 2021;10(14):23.

15. Eastment MC, Kinuthia J, Wang L, Wanje G, Wilson K, Kaggiah A, et al. Late antiretroviral refills and condomless sex in a cohort of HIV-seropositive pregnant and postpartum Kenyan women. Plos One. 2021;16(7):14.

16. Chalmers BP, Lebowitz JS, Chiu YF, Joseph AD, Padgett DE, Bostrom MPG, et al. Changes in opioid discharge prescriptions after primary total hip and total knee arthroplasty affect opioid refill rates and morphine milligram equivalents AN INSTITUTIONAL EXPERIENCE OF 20,000 PATIENTS. Bone Joint J. 2021;103B(7):103-10.

17. Waughtal J, Luong P, Sandy L, Chavez C, Ho PM, Bull S. Nudge me: tailoring text messages for prescription adherence through N-of-1 interviews. Transl Behav Med. 2021;11(10):1832-8.

18. Ojinnaka CO, Adepoju OE. Racial and Ethnic Disparities in Health Information Technology Use and Associated Trends among Individuals Living with Chronic Diseases. Popul Health Manag. 2021;24(6):675-80.

19. Massie L, Gunaseelan V, Waljee J, Brummett C, Schwalb JM. Relationship between initial opioid prescription size and likelihood of refill after spine surgery. Spine Journal. 2021;21(5):772-8.

20. Subbaraman R, Thomas BE, Kumar JV, Thiruvengadam K, Khandewale A, Kokila S, et al. Understanding Nonadherence to Tuberculosis Medications in India Using Urine Drug Metabolite Testing: A Cohort Study. Open Forum Infect Dis. 2021;8(6):9.

21. Luong P, Glorioso TJ, Grunwald GK, Peterson P, Allen LA, Khanna A, et al. Text Message Medication Adherence Reminders Automated and Delivered at Scale Across Two Institutions Testing the Nudge System: Pilot Study. Circ-Cardiovasc Qual Outcomes. 2021;14(5):8.

22. Joret R, Matti N, Beck M, Michel B. Medication adherence and persistence among patients with non-small cell lung cancer receiving tyrosine kinase inhibitors and estimation of the economic burden associated with the unused medicines. J Oncol Pharm Pract.10.

23. Asieba IO, Oqua DA, Wutoh AA, Agu KA, Omeh OI, Adeyanju ZA, et al. Antiretroviral therapy in community pharmacies - Implementation and outcomes of a differentiated drug delivery model in Nigeria. Res Soc Adm Pharm. 2021;17(5):842-9.

24. Craig LS, Peacock E, Mohundro BL, Silver JH, Marsh J, Johnson TC, et al. Implicit and Explicit Attitudes Toward Antihypertensive Medications Explain Variation in Pharmacy Refill and Self-Reported Adherence Beyond Traditional Risk Factors: Potential Novel Mechanism Underlying Adherence. J Am Heart Assoc. 2021;10(6):13.

25. Hecht S, Halstead NV, Boxley P, Brockel MA, Rove KO. Opioid prescribing patterns following implementation of Enhanced Recovery After Surgery (ERAS) protocol in pediatric patients undergoing lower tract urologic reconstruction. J Pediatr Urol. 2021;17(1):8.

26. Ibiloye O, Akande P, Plang J, Emerenini F, Omole T, Osindero O, et al. Community health worker-led ART delivery improved scheduled antiretroviral drug refill among men who have sex with men in Lagos State, Nigeria. Int Health. 2021;13(2):196-8.

27. Magny-Normilus C, Nolido NV, Borges JC, Brady M, Labonville S, Williams D, et al. Effects of an Intensive Discharge Intervention on Medication Adherence, Glycemic Control, and Readmission Rates in Patients With Type 2 Diabetes. J Patient Saf. 2021;17(2):73-80.

28. Meza BC, Swarup I, Woodard T, Cazzulino A, Talwar D, Shah AS. Refilling Opioid Prescriptions After Pediatric Orthopaedic Surgery: An Analysis of Incidence and Risk Factors. J Pediatr Orthop. 2021;41(3):E291-E5.

29. Tessua KK, Munseri P, Matuja SS. Outcomes within a year following first ever stroke in Tanzania. Plos One. 2021;16(2):13.

30. Fenelon-Dimanche R, Guenette L, Trudel-Bourgault F, Yousif A, Lalonde G, Beauchesne MF, et al. Development of an electronic tool (e-AdPharm) to address unmet needs and barriers of community pharmacists to provide medication adherence support to patients. Res Soc Adm Pharm. 2021;17(3):506-13.

31. Lim PC, Chung YY, Tan SJ, Wong T, Permalu DD, Cheah TK, et al. Comparing the cost, glycaemic control and medication adherence of utilizing patients' own medicines (POMs) versus usual dispensing among diabetic patients in an outpatient setting. Daru. 2021;29(1):125-32.

32. Abd-Alrazaq A, Safi Z, Bewick BM, Househ M, Gardner PH. Patients' Perspectives About Factors Affecting Their Use of Electronic Personal Health Records in England: Qualitative Analysis. J Med Internet Res. 2021;23(1):13.

33. Stambough JB, Hui R, Siegel ER, Edwards PK, Barnes CL, Mears SC. Narcotic Refills and Patient Satisfaction With Pain Control After Total Joint Arthroplasty. J Arthroplast. 2021;36(2):454-61.

34. Erker R, Alefan Q, Goodridge D, Crawley A, Rabbitskin N, Bighead S, et al. Evaluation of a medication safety and adherence program within a First Nations community in Saskatchewan, Canada. J Am Pharm Assoc. 2021;61(1):E39-E45.

35. Criner GJ, Cole T, Hahn KA, Kastango K, Eudicone J, Gilbert I. The Impact of Budesonide/Formoterol pMDI Medication Reminders on Adherence in Chronic Obstructive Pulmonary Disease (COPD) Patients: Results of a Randomized, Phase 4, Clinical Study. Int J Chronic Obstr Pulm Dis. 2021;16:563-77.

36. Plaza V, Giner J, Curto E, Alonso-Ortiz MB, Orue MI, Vega JM, et al. Assessing Adherence by Combining the Test of Adherence to Inhalers With Pharmacy Refill Records. J Invest Allergol Clin Immunol. 2021;31(1):58-64.

37. Cassidy T, Grimsrud A, Keene C, Lebelo K, Hayes H, Orrell C, et al. Twenty-four-month outcomes from a cluster-randomized controlled trial of extending antiretroviral therapy refills in ART adherence clubs. J Int AIDS Soc. 2020;23(12):10.

38. Solouki S, Vega M, Agalliu I, Abraham NE. Patient Satisfaction and Refill Rates After Decreasing Opioids Prescribed for Urogynecologic Surgery. Female Pelvic Med Reconstr Surg. 2020;26(12):E78-E82.

39. Omonaiye O, Nicholson P, Kusljic S, Mohebbi M, Manias E. Medication-based Refill Adherence Among Pregnant Women Living With HIV in Nigeria. Clin Ther. 2020;42(11):E209-E19.

40. Schlosser EG, Neff E, Francis SM, Vormohr H, Hincapie AL. Implementation of a protocol-driven pharmacy technician refill process at a large physician network. J Am Pharm Assoc. 2020;60(6):E341-E8.

41. Vordenberg SE, Zikmund-Fisher BJ. Older adults' strategies for obtaining medication refills in hypothetical scenarios in the face of COVID-19 risk. J Am Pharm Assoc. 2020;60(6):915-+.

42. Meiqari L, Nguyen TPL, Essink D, Wright P, Scheele F. Strengthening human and physical infrastructure of primary healthcare settings to deliver hypertension care in Vietnam: a mixed-methods comparison of two provinces. Health Policy Plan. 2020;35(8):918-30.

43. Hart LC, Grosse SD, Danielson ML, Baum RA, Kemper AR. Changes in Provider Type and Prescription Refills Among Privately Insured Children and Youth With ADHD. J Atten Disord. 2021;25(14):2028-36.

44. Biederman J, Fried R, DiSalvo M, Driscoll H, Green A, Biederman I, et al. A novel digital health intervention to improve patient engagement to stimulants in adult ADHD in the primary care setting: Preliminary findings from an open label study. Psychiatry Res. 2020;291:7.

45. Kern DM, Cepeda MS, Salas M, Phillips S, Secrest MH, Wedin GP. Frequency of Early Refills for Opioids in the United States. Pain Med. 2020;21(9):1818-24.

46. Khazi ZM, Baron J, Shamrock A, Gulbrandsen T, Bedard N, Wolf B, et al. Preoperative Opioid Usage, Male Sex, and Preexisting Knee Osteoarthritis Impacts Opioid Refills After Isolated Arthroscopic Meniscectomy: A Population-Based Study. Arthroscopy. 2020;36(9):2478-85.

47. de Bouille JG, Collignon M, Capsec J, Guillon L, Le Moal G, Barin F, et al. Low-level HIV viremia is associated with low antiretroviral prescription refill rates and social deprivation. Aids Care-Psychol Socio-Med Asp Aids-Hiv. 2021;33(11):1445-50.

48. Kirsch M, Montgomery JR, Hu HM, Englesbe M, Hallstrom B, Brummett CM, et al. Association between insurance cost-sharing subsidy and postoperative opioid prescription refills among Medicare patients. Surgery. 2020;168(2):244-52.

49. Ung D, Dalli LL, Lopez D, Sanfilippo FM, Kim J, Andrew NE, et al. Assuming one dose per day yields a similar estimate of medication adherence in patients with stroke: An exploratory analysis using linked registry data. Br J Clin Pharmacol. 2021;87(3):1089-97.

50. Fatti G, Ngorima-Mabhena N, Mothibi E, Muzenda T, Choto R, Kasu T, et al. Outcomes of Three- Versus Six-Monthly Dispensing of Antiretroviral Treatment (ART) for Stable HIV Patients in Community ART Refill Groups: A Cluster-Randomized Trial in Zimbabwe. Jaids. 2020;84(2):162-72.

51. Pascoe SJS, Scott NA, Fong RM, Murphy J, Huber AN, Moolla A, et al. "Patients are not the same, so we cannot treat them the same" - A qualitative content analysis of provider, patient and implementer perspectives on differentiated service delivery models for HIV treatment in South Africa. J Int AIDS Soc. 2020;23(6):19.

52. Gong WJ, Zhang C, An D, Xiao SY, Yu Y, Caine ER. The association between a free medicine program and functioning in people with schizophrenia: a cross-sectional study in Liuyang, China. Peerj. 2020;8:12.

53. Galozy A, Nowaczyk S, Sant'Anna A, Ohlsson M, Lingman M. Pitfalls of medication adherence approximation through EHR and pharmacy records: Definitions, data and computation. Int J Med Inform. 2020;136:13.

54. Ramirez-Zohfeld V, Seltzer A, Xiong LD, Morse L, Lindquist LA. Use of Electronic Health Records by Older Adults, 85 Years and Older, and Their Caregivers. J Am Geriatr Soc. 2020;68(5):1078-82.

55. Blackburn DF, Evans C, Mansell H, Taylor J, Jorgenson D, Mansell K, et al. Impact of the SIMPL-SYNC Refill Synchronization Program on medication adherence: A pragmatic randomized controlled trial. J Am Pharm Assoc. 2020;60(2):328-+.

56. Lapidus JB, Santosa KB, Skolnick GB, Som A, Cho GJ, Waljee JF, et al. Opioid Prescribing and Use Patterns in Postsurgical Facial Trauma Patients. Plast Reconstr Surg. 2020;145(3):780-9.

57. Fried R, DiSalvo M, Kelberman C, Adler A, McCafferty D, Woodworth KY, et al. An innovative SMS intervention to improve adherence to stimulants in children with ADHD: Preliminary findings. J Psychopharmacol. 2020;34(8):883-90.

58. Chiu PE, Tsao HM, Tsai CH. Discrepancy Among Self-Reported Adherence, Prescription Refills, and Actual Anticoagulant Control. J Nurs Res. 2020;28(1):8.

59. Hesso I, Gebara SN, Greene G, Stello RWC, Kayyali R. A quantitative evaluation of adherence and inhalation technique among respiratory patients: An observational study using an electronic inhaler assessment device. Int J Clin Pract. 2020;74(2):11.

60. Moucheraud C, Hing M, Seleman J, Phiri K, Chibwana F, Kahn D, et al. Integrated care experiences and out-of-pocket expenditures: a cross-sectional survey of adults receiving treatment for HIV and hypertension in Malawi. BMJ Open. 2020;10(2):9.

61. Chen YJ, Chang J, Yang SY. Psychometric Evaluation of Chinese Version of Adherence to Refills and Medications Scale (ARMS) and Blood-Pressure Control Among Elderly with Hypertension. Patient Prefer Adherence. 2020;14:213-20.

62. Degli Esposti L, Buda S, Nappi C, Paoli D, Perrone V. Implications of COVID-19 Infection on Medication Adherence with Chronic Therapies in Italy: A Proposed Observational Investigation by the Fail-to-Refill Project. Risk Manag Healthc Policy. 2020;13:3179-85.

63. Lillis S, Lack L. Repeat prescribing policy in New Zealand general practice: making it better. J Prim Health Care. 2020;12(4):373-6.

64. Merola D, Calotta NA, Lu ZA, Lifchez SD, Aliu O, Coon D. Initial Opioid Prescriptions Predict Continued Narcotic Use: Analysis of 24,594 Reduction Mammaplasty Patients. Plast Reconstr Surg. 2020;145(1):20-30.

65. Lee JZ, Pasha AK, Glasgow AE, Habermann EB, Kusumoto FM, McLeod CJ, et al. Postoperative opioid prescription patterns and new opioid refills following cardiac implantable electronic device procedures. Heart Rhythm. 2019;16(12):1841-8.

66. Prayaga RB, Agrawal R, Nguyen B, Jeong EW, Noble HK, Paster A, et al. Impact of Social Determinants of Health and Demographics on Refill Requests by Medicare Patients Using a Conversational Artificial Intelligence Text Messaging Solution: Cross-Sectional Study. Jmir Mhealth and Uhealth. 2019;7(11):15.

67. Chiu AS, Freedman-Weiss MR, Jean RA, Cohen E, Yoo PS. No refills: The durable impact of a multifaceted effort by surgical trainees to minimize the prescription of postoperative opioids. Surgery. 2019;166(5):758-63.

68. Linder BJ, Occhino JA, Wiest SR, Klingele CJ, Trabuco EC, Gebhart JB. Assessing the impact of procedure-specific opioid prescribing recommendations on opioid stewardship following pelvic organ prolapse surgery. Am J Obstet Gynecol. 2019;221(5):8.

69. Goplen CM, Randall JR, Kang SH, Vakilian F, Jones CA, Voaklander DC, et al. The Influence of Allowable Refill Gaps on Detecting Long-Term Opioid Therapy: An Analysis of Population-Based Administrative Dispensing Data Among Patients with Knee Arthritis Awaiting Total Knee Arthroplasty. J Manag Care Spec Pharm. 2019;25(10):1064-+.

70. Mantell JE, Masvawure TB, Mapingure M, Apollo T, Gwanzura C, Block L, et al. Engaging men in HIV programmes: a qualitative study of male engagement in community-based antiretroviral refill groups in Zimbabwe. J Int AIDS Soc. 2019;22(10):11.

71. Dillon P, McDowell R, Smith SM, Gallagher P, Cousins G. Determinants of intentions to monitor antihypertensive medication adherence in Irish community pharmacy: a factorial survey. BMC Fam Pract. 2019;20(1):14.

72. Elsamadicy AA, Charalambous L, Adif SM, Drysdale N, Lee M, Koo AB, et al. Reduced Influence of Affective Disorders on 6-Week and 3-Month Narcotic Refills After Primary Complex Spinal Fusions for Adult Deformity Correction: A Single-Institutional Study. World Neurosurg. 2019;129:E311-E6.

73. Lillis S, Macklin N, Thorn M, Wicks E, Good K, Lack L, et al. Repeat prescribing safety survey. J Prim Health Care. 2019;11(3):243-8.

74. Amstutz A, Lejone TI, Khesa L, Muhairwe J, Nsakala BL, Tlali K, et al. VIBRA trial - Effect of village-based refill of ART following home-based same-day ART initiation vs clinic-based ART refill on viral suppression among individuals living with HIV: protocol of a cluster-randomized clinical trial in rural Lesotho. Trials. 2019;20(1):14.

75. Neuner JM, Frgestrom NM, Laud PW, Nattinger AB, Beyer KMM, Flynn KE, et al. The association of pharmacy fill synchronization with breast cancer endocrine therapy adherence. Cancer. 2019;125(22):3960-5.

76. Bochner AF, Meacham E, Mhungu N, Manyanga P, Petracca F, Muserere C, et al. The rollout of Community ART Refill Groups in Zimbabwe: a qualitative evaluation. J Int AIDS Soc. 2019;22(8):7.

77. Genn L, Chapman J, Okatch H, Abell N, Marukutira T, Tshume O, et al. Pharmacy Refill Data are Poor Predictors of Virologic Treatment Outcomes in Adolescents with HIV in Botswana. AIDS Behav. 2019;23(8):2130-7.

78. Yildirim C, Garvie PA, Chernoff M, Wilkins ML, Patton ED, Williams PL, et al. The Role of Pharmacy Refill Measures in Assessing Adherence and Predicting HIV Disease Markers in Youth with Perinatally-Acquired HIV (PHIV). AIDS Behav. 2019;23(8):2109-20.

79. Wilkinson L, Grimsrud A, Cassidy T, Orrell C, Voget J, Hayes H, et al. A cluster randomized controlled trial of extending ART refill intervals to six-monthly for anti-retroviral adherence clubs. BMC Infect Dis. 2019;19:9.

80. Biederman J, Fried R, DiSalvo M, Woodworth KY, Biederman I, Noyes E, et al. A Novel Text Message Intervention to Improve Adherence to Stimulants in Adults With Attention Deficit/Hyperactivity Disorder. J Clin Psychopharmacol. 2019;39(4):351-6.

81. Demik DE, Rojas EO, Anthony CA, Bedard NA, Dowdle SB, Bollier M, et al. Opioid Prescription Refills After Osteochondral Procedures of the Knee. Arthroscopy. 2019;35(7):2083-8.

82. Wilke BK, Foster DT, Roberts CA, Shi GG, Lesser ER, Heckman MG, et al. Early In-Hospital Pain Control Is a Stronger Predictor for Patients Requiring a Refill of Narcotic Pain Medication Compared to the Amount of Narcotics Given at Discharge. J Arthroplast. 2019;34(7):1354-8.

83. Szatmari S, Ajtay A, Balint M, Takats A, Oberfrank F, Bereczki D. Linking Individual Patient Data to Estimate Incidence and Prevalence of Parkinson's Disease by Comparing Reports of Neurological Services and Pharmacy Prescription Refills at a Nationwide Level. Front Neurol. 2019;10:8.

84. Dillon P, Smith SM, Gallagher PJ, Cousins G. Association between gaps in antihypertensive medication adherence and injurious falls in older community-dwelling adults: a prospective cohort study. BMJ Open. 2019;9(3):9.

85. Karlsson SA, Eliasson B, Franzen S, Miftaraj M, Svensson AM, Sundell KA. Risk of cardiovascular event and mortality in relation to refill and guideline adherence to lipid-lowering medications among patients with type 2 diabetes mellitus in Sweden. BMJ Open Diab Res Care. 2019;7(1):13.

86. Neuner JM, Fergestrom N, Laud PW, Pezzin L. Factors Influencing Prescription Drug Synchronization: The Complex Role of Number of Medications. J Manag Care Spec Pharm. 2019;25(6):714-8.

87. Allemann SS, Dediu D, Dima AL. Beyond Adherence Thresholds: A Simulation Study of the Optimal Classification of Longitudinal Adherence Trajectories From Medication Refill Histories. Front Pharmacol. 2019;10:13.

88. Lee Y, Park YR, Lee JS, Lee SB, Chung IY, Son BH, et al. Prescription Refill Gap of Endocrine Treatment from Electronic Medical Records as a Prognostic Factor in Breast Cancer Patients. J Breast Canc. 2019;22(1):86-95.

89. Kurata K, Onuki M, Yoshizumi K, Taniai E, Dobashi A. Proportion of Japanese outpatients filling prescriptions for long-term medication regimens. Patient Prefer Adherence. 2019;13:667-73.

90. Lee JS, Howard RA, Klueh MP, Englesbe MJ, Waljee JF, Brummett CM, et al. The Impact of Education and Prescribing Guidelines on Opioid Prescribing for Breast and Melanoma Procedures. Ann Surg Oncol. 2019;26(1):17-24.

91. Casillas A, Moreno G, Grotts J, Tseng CH, Morales LS. A Digital Language Divide? The Relationship between Internet Medication Refills and Medication Adherence among Limited English Proficient (LEP) Patients. J Racial Ethn Health Disparities. 2018;5(6):1373-80.

92. Ermes M, Vuorinen AL, Schrader G, Bidargaddi N. Correlation between symptoms and functioning in psychiatric patients and temporal patterns of medication refills derived from pharmacy prescription claims. Australas Psychiatry. 2018;26(6):643-7.

93. Deeds S, Carr S, Garrison M, Fainstad T. Delivery of Standardized Patient Instructions in the After-Visit Summary Reduces Telephone Calls Between Clinic Visits. Am J Med Qual. 2018;33(6):642-8.

94. Williams LG, Peacock E, Joyce C, Bazzano LA, Sarpong D, Whelton PK, et al. Risk Factors for Low Pharmacy Refill Adherence Among Older Hypertensive Men and Women by Race. Am J Med Sci. 2018;356(5):464-75.

95. Pasipamire L, Nesbitt RC, Ndlovu S, Sibanda G, Mamba S, Lukhele N, et al. Retention on ART and predictors of disengagement from care in several alternative community-centred ART refill models in rural Swaziland. J Int AIDS Soc. 2018;21(9):9.

96. VanEpps EM, Troxel AB, Villamil E, Saulsgiver KA, Zhu JS, Chin JY, et al. Financial Incentives for Chronic Disease Management: Results and Limitations of 2 Randomized Clinical Trials with New York Medicaid Patients. Am J Health Promot. 2018;32(7):1537-43.

97. Sekhri S, Arora NS, Cottrell H, Baerg T, Duncan A, Hu HM, et al. Probability of Opioid Prescription Refilling After Surgery Does Initial Prescription Dose Matter? Ann Surg. 2018;268(2):271-6.

98. Hernandez NM, Parry JA, Mabry TM, Taunton MJ. Patients at Risk: Preoperative Opioid Use Affects Opioid Prescribing, Refills, and Outcomes After Total Knee Arthroplasty. J Arthroplast. 2018;33(7):S142-S6.

99. Gildon BL, John B, Condren M, Keast S, Maglunog R, Johnson JL, et al. Pharmacist-managed short-acting beta agonist refill service in a general pediatric clinic. J Am Pharm Assoc. 2018;58(3):296-302.

100. Hofmeyer BA, Look KA, Hager DR. Refill-Based Medication Use Quality Measures in Kidney Transplant Recipients: Examination of Proportion of Days Covered and Medication Possession Ratio. J Manag Care Spec Pharm. 2018;24(4):367-72.

101. King S, Miani C, Exley J, Larkin J, Kirtley A, Payne RA. Impact of issuing longer- versus shorter-duration prescriptions: a systematic review. Br J Gen Pract. 2018;68(669):E286-E92.

102. Barry A, Ford N, El-Khatib Z. Factors for incomplete adherence to antiretroviral therapy including drug refill and clinic visits among older adults living with human immunodeficiency virus - cross-sectional study in South Africa. Trop Med Int Health. 2018;23(3):270-8.

103. Karlsson SA, Hero C, Svensson AM, Franzen S, Miftaraj M, Gudbjornsdottir S, et al. Association between refill adherence to lipid-lowering medications and the risk of cardiovascular disease and mortality in Swedish patients with type 2 diabetes mellitus: a nationwide cohort study. BMJ Open. 2018;8(3):14.

104. Wu YP, Stenehjem DD, Linder LA, Yu B, Parsons BG, Mooney R, et al. Adherence to Oral Medications During Maintenance Therapy Among Children and Adolescents With Acute Lymphoblastic Leukemia: A Medication Refill Analysis. J Pediatr Oncol Nurs. 2018;35(2):86-93.

105. Husain FA, Hollis HW, Pottorf BJ, Rogers JL, Golembeski SM, Johnson JM. The Effect of Transoral Gastric Remnant Extraction on Prescription Opioid Refills and Surgical Site Infections in Patients Undergoing Sleeve Gastrectomy. Bariatr Surg Pract Patient Care. 2018;13(1):12-7.

106. Rim MH, Thomas KC, Hatch B, Kelly M, Tyler LS. Development and implementation of a centralized comprehensive refill authorization program in an academic health system. Am J Health-Syst Pharm. 2018;75(3):132-8.

107. Fatti G, Ngorima-Mabhena N, Chirowa F, Chirwa B, Takarinda K, Tafuma TA, et al. The effectiveness and cost-effectiveness of 3-vs. 6-monthly dispensing of antiretroviral treatment (ART) for stable HIV patients in community ART-refill groups in Zimbabwe: study protocol for a pragmatic, cluster-randomized trial. Trials. 2018;19:10.

108. Avong YK, Aliyu GG, Jatau B, Gurumnaan R, Danat N, Kayode GA, et al. Integrating community pharmacy into community based anti-retroviral therapy program: A pilot implementation in Abuja, Nigeria. Plos One. 2018;13(1):10.

109. Arnet I, Greenland M, Knuiman MW, Rankin JM, Hung J, Nedkoff L, et al. Operationalization and validation of a novel method to calculate adherence to polypharmacy with refill data from the Australian pharmaceutical benefits scheme (PBS) database. Clin Epidemiol. 2018;10:181-94.

110. Biset N, Lelubre M, Senterre C, Amighi K, Bugnon O, Schneider MP, et al. Assessment of medication adherence and responsible use of isotretinoin and contraception through Belgian community pharmacies by using pharmacy refill data. Patient Prefer Adherence. 2018;12:153-61.

111. Jennings AA, Guerin N, Foley T. Development of a tool for monitoring the prescribing of antipsychotic medications to people with dementia in general practice: a modified eDelphi consensus study. Clin Interv Aging. 2018;13:2107-17.

112. Lomper K, Chabowski M, Chudiak A, Bialoszewski A, Dudek K, Jankowska-Polanska B. Psychometric evaluation of the Polish version of the Adherence to Refills and Medications Scale (ARMS) in adults with hypertension. Patient Prefer Adherence. 2018;12:2661-70.

113. de Llano LP, Sanmartin AP, Gonzalez-Barcala FJ, Mosteiro-Anon M, Abelaira DC, Quintas RD, et al. Assessing adherence to inhaled medication in asthma: Impact of once-daily versus twice-daily dosing frequency. The ATAUD study. J Asthma. 2018;55(9):933-8.

114. Prayaga RB, Jeong EW, Feger E, Noble HK, Kmiec M, Prayaga RS. Improving Refill Adherence in Medicare Patients With Tailored and Interactive Mobile Text Messaging: Pilot Study. Jmir Mhealth and Uhealth. 2018;6(1):10.

115. Been SK, Yildiz E, Nieuwkerk PT, Pogany K, van de Vijver D, Verbon A. Self-reported adherence and pharmacy refill adherence are both predictive for an undetectable viral load among HIV-infected migrants receiving cART. Plos One. 2017;12(11):12.

116. Mueller T, Alvarez-Madrazo S, Robertson C, Bennie M. Use of direct oral anticoagulants in patients with atrial fibrillation in Scotland: Applying a coherent framework to drug utilisation studies. Pharmacoepidemiol Drug Saf. 2017;26(11):1378-86.

117. Gokdogan F, Kes D. Validity and reliability of the Turkish Adherence to Refills and Medications Scale. Int J Nurs Pract. 2017;23(5):7.

118. Karlsson SA, Hero C, Eliasson B, Franzen S, Svensson AM, Miftaraj M, et al. Refill adherence and persistence to lipid-lowering medicines in patients with type 2 diabetes: A nation-wide register-based study. Pharmacoepidemiol Drug Saf. 2017;26(10):1220-32.

119. Girdish C, Shrank W, Freytag S, Chen D, Gebhard D, Bunton A, et al. The impact of a retail prescription synchronization program on medication adherence. J Am Pharm Assoc. 2017;57(5):579-+.

120. Witry MJ, Guirguis LM. Any questions? Yes, do pharmacists monitor medications at refill? J Am Pharm Assoc. 2017;57(5):591-5.

121. Andrews SB, Marcy TR, Osborn B, Planas LG. The impact of an appointment-based medication synchronization programme on chronic medication adherence in an adult community pharmacy population. J Clin Pharm Ther. 2017;42(4):461-6.

122. Price J, Man SL, Bartlett S, Taylor K, Dinwoodie M, Bowie P. Repeat prescribing of medications: A system-centred risk management model for primary care organisations. J Eval Clin Pract. 2017;23(4):779-96.

123. Prust ML, Banda CK, Nyirenda R, Chimbwandira F, Kalua T, Jahn A, et al. Multi-month prescriptions, fast-track refills, and community ART groups: results from a process evaluation in Malawi on using differentiated models of care to achieve national HIV treatment goals. J Int AIDS Soc. 2017;20:10.

124. Martin D, Luz PM, Lake JE, Clark JL, Campos DP, Veloso VG, et al. Pharmacy refill data can be used to predict virologic failure for patients on antiretroviral therapy in Brazil. J Int AIDS Soc. 2017;20:5.

125. Kovacic L, de Haan N, de Lemos ML, Schaff K, Walisser S. Adherence with capecitabine: A population-based analysis based on prescription refill data. J Oncol Pharm Pract. 2017;23(4):284-7.

126. Hui RL, Adams AL, Niu F, Ettinger B, Yi DK, Chandra M, et al. Predicting Adherence and Persistence with Oral Bisphosphonate Therapy in an Integrated Health Care Delivery System. J Manag Care Spec Pharm. 2017;23(4):503-12.

127. Samson CM, Mager D, Frazee S, Yu F. Remission in Pediatric Inflammatory Bowel Disease Correlates With Prescription Refill Adherence Rates. J Pediatr Gastroenterol Nutr. 2017;64(4):575-9.

128. McInnes DK, Shimada SL, Midboe AM, Nazi KM, Zhao SB, Wu J, et al. Patient Use of Electronic Prescription Refill and Secure Messaging and Its Association With Undetectable HIV Viral Load: A Retrospective Cohort Study. J Med Internet Res. 2017;19(2):13.

129. Doshi JA, Lim R, Li PX, Young PP, Lawnicki VF, Troxel AB, et al. Synchronized Prescription Refills and Medication Adherence: A Retrospective Claims Analysis. Am J Manag Care. 2017;23(2):98-+.

130. Rosen CS, Azevedo KJ, Tiet QQ, Greene CJ, Wood AE, Calhoun P, et al. An RCT of Effects of Telephone Care Management on Treatment Adherence and Clinical Outcomes Among Veterans With PTSD. Psychiatr Serv. 2017;68(2):151-8.

131. Adetunji AA, Muyibi SA, Imhansoloeva M, Ibraheem OM, Sunmola A, Kolawole OO, et al. Mobile phone use for a social strategy to improve antiretroviral refill experience at a low-resource HIV clinic: patient responses from Nigeria. Aids Care-Psychol Socio-Med Asp Aids-Hiv. 2017;29(5):575-8.

132. Alzuman SN, Al-Humaidan AS. Clinical Pharmacist interventions in Refill Clinic at Tertiary Care Eye Specialist Hospital. Saudi Pharm J. 2017;25(1):70-5.

133. Comer D, Mearns E, Olivere L, Elliott DJ. Primary Care Provider Perspectives on Electronic Medication Refill History. Am J Med Qual. 2017;32(1):43-7.

134. Coppock D, Zambo D, Moyo D, Tanthuma G, Chapman J, Lo Re V, et al. Development and Usability of a Smartphone Application for Tracking Antiretroviral Medication Refill Data for Human Immunodeficiency Virus. Methods Inf Med. 2017;56(5):351-9.

135. Ford H, Trent S, Wickizer S. Pharmacy services after a tank car derailment and toxic chemical release in Blount County, Tennessee. J Am Pharm Assoc. 2017;57(1):56-+.

136. Mekuria LA, Prins JM, Yalew AW, Sprangers MAG, Nieuwkerk PT. Sub-optimal adherence to combination anti-retroviral therapy and its associated factors according to self-report, clinician-recorded and pharmacy-refill assessment methods among HIV-infected adults in Addis Ababa. Aids Care-Psychol Socio-Med Asp Aids-Hiv. 2017;29(4):428-35.

137. Moran C, Doyle F, Sulaiman I, Bennett K, Greene G, Molloy GJ, et al. The INCA (TM) (Inhaler Compliance Assessment (TM)): A comparison with established measures of adherence. Psychol Health. 2017;32(10):1266-87.

138. Liu XY, Kubilis P, Bussing R, Winterstein AG. Development of a refill pattern method to measure polypharmacy in administrative claims databases. Pharmacoepidemiol Drug Saf. 2016;25(12):1407-13.

139. Siproudhis J, Chapron A, Allory E, Fiquet L, Dauguet S, Oger E. Patient's preferences for repeat prescriptions in general practice. Therapie. 2016;71(6):579-87.

140. Blackburn DF, Tran D, Quiring C. Evaluation of a refill synchronization program in two community pharmacies. J Am Pharm Assoc. 2016;56(6):656-9.

141. Lester CA, Look KA, Chui MA. Is the Currently Used Prescription Adjudication Date a Good Proxy for Calculating Medication Refill Adherence? J Manag Care Spec Pharm. 2016;22(11):1311-7.

142. Lyons I, Barber N, Raynor DK, Wei L. The Medicines Advice Service Evaluation (MASE): a randomised controlled trial of a pharmacist-led telephone based intervention designed to improve medication adherence. BMJ Qual Saf. 2016;25(10):759-69.

143. Hayashi K, Wood E, Kerr T, Dong HR, Nguyen P, Puskas CM, et al. Factors associated with optimal pharmacy refill adherence for antiretroviral medications and plasma HIV RNA non-detectability among HIV-positive crack cocaine users: a prospective cohort study. BMC Infect Dis. 2016;16:12.

144. Doshi JA, Lim R, Li PX, Young PP, Lawnicki VF, State JJ, et al. A Synchronized Prescription Refill Program Improved Medication Adherence. Health Aff. 2016;35(8):1504-12.

145. Harrison TN, Green KR, Liu ILA, Vansomphone SS, Handler J, Scott RD, et al. Automated Outreach for Cardiovascular-Related Medication Refill Reminders. J Clin Hypertens. 2016;18(7):641-6.

146. Kumar JB, Bosworth HB, Sleath B, Woolson S, Olsen M, Danus S, et al. Quantifying Glaucoma Medication Adherence: The Relationship Between Self-Report, Electronic Monitoring, and Pharmacy Refill. J Ocular Pharmacol Ther. 2016;32(6):346-54.

147. Lester CA, Mott DA, Chui MA. The Influence of a Community Pharmacy Automatic Prescription Refill Program on Medicare Part D Adherence Metrics. J Manag Care Spec Pharm. 2016;22(7):801-7.

148. Lester CA, Chui MA. The prescription pickup lag, an automatic prescription refill program, and community pharmacy operations. J Am Pharm Assoc. 2016;56(4):427-32.

149. Mekuria LA, Prins JM, Yalew AW, Sprangers MAG, Nieuwkerk PT. Which adherence measure - self-report, clinician recorded or pharmacy refill - is best able to predict detectable viral load in a public ART programme without routine plasma viral load monitoring? Trop Med Int Health. 2016;21(7):856-69.

150. Davidow AL, Thomas P, Kim S, Passannante M, Tsai S, Tan C. Access to Care in the Wake of Hurricane Sandy, New Jersey, 2012. Dis Med Public Health Prep. 2016;10(3):485-91.

151. Bjerkeli PJ, Jonsson AK, Lesen E, Mardby AC, Sundell KA. Refill Adherence in Relation to Substitution and the Use of Multiple Medications: A Nationwide Population Based Study on New ACE-Inhibitor Users. Plos One. 2016;11(5):15.

152. Bushnell GA, Sturmer T, White A, Pate V, Swanson SA, Azrael D, et al. Predicting persistence to antidepressant treatment in administrative claims data: Considering the influence of refill delays and prior persistence on other medications. J Affect Disord. 2016;196:138-47.

153. Bonacci RA, Frasca K, Swift L, Sha DH, Bilker WB, Bamford L, et al. Antiretroviral Refill Adherence Correlates with, But Poorly Predicts Retention in HIV Care. AIDS Behav. 2016;20(5):1060-7.

154. Kim CJ, Park E, Schlenk EA, Kim M, Kim DJ. Psychometric Evaluation of a Korean Version of the Adherence to Refills and Medications Scale (ARMS) in Adults With Type 2 Diabetes. Diabetes Educ. 2016;42(2):188-98.

155. Lyles CR, Sarkar U, Schillinger D, Ralston JD, Allen JY, Nguyen R, et al. Refilling medications through an online patient portal: consistent improvements in adherence across racial/ethnic groups. J Am Med Inf Assoc. 2016;23(E1):E28-E33.

156. Moreno G, Lin EH, Chang E, Johnson RL, Berthoud H, Solomon CC, et al. Disparities in the Use of Internet and Telephone Medication Refills among Linguistically Diverse Patients. J Gen Intern Med. 2016;31(3):282-8.

157. Schoenhaus R, Lustig A, Rivas S, Monrreal V, Westrich KD, Dubois RW. Using an Electronic Medication Refill System to Improve Provider Productivity in an Accountable Care Setting. J Manag Care Spec Pharm. 2016;22(3):204-8.

158. Grant S, Mesman J, Guthrie B. Spatio-temporal elements of articulation work in the achievement of repeat prescribing safety in UK general practice. Sociol Health Ill. 2016;38(2):306-24.

159. Lopez A, Long C, Happe LE, Relish M. Using Sequence Discovery to Target Outreach for Diabetes Medication Adherence. Am J Manag Care. 2015;21(11):E601-+.

160. Matlin OS, Kymes SM, Averbukh A, Choudhry NK, Brennan TA, Bunton A, et al. Community Pharmacy Automatic Refill Program Improves Adherence to Maintenance Therapy and Reduces Wasted Medication. Am J Manag Care. 2015;21(11):785-91.

161. Nosyk B, Min JE, Colley G, Lima VD, Yip B, Milloy MJS, et al. The causal effect of opioid substitution treatment on HAART medication refill adherence. Aids. 2015;29(8):965-73.

162. Hansen RA, Voils CI, Farley JF, Powers BJ, Sanders LL, Sleath B, et al. Prescriber Continuity and Medication Adherence for Complex Patients. Ann Pharmacother. 2015;49(3):293-302.

163. Stanek JJ, Renslow MA, Kalliainen LK. The Effect of an Educational Program on Opioid Prescription Patterns in Hand Surgery: A Quality Improvement Program. J Hand Surg-Am Vol. 2015;40(2):341-6.

164. Kang H, Yip B, Chau W, De La Rosa AN, Hall D, Barrios R, et al. Continuing professional development in HIV chronic disease management for primary care providers. Med Teach. 2015;37(8):714-7.

165. Trusell H, Sundell KA. Effects of generic substitution on refill adherence to statin therapy: a nationwide population-based study. BMC Health Serv Res. 2014;14:8.

166. Witry MJ, Doucette WR. Community pharmacists, medication monitoring, and the routine nature of refills: A qualitative study. J Am Pharm Assoc. 2014;54(6):594-U121.

167. Sangeda RZ, Mosha F, Prosperi M, Aboud S, Vercauteren J, Camacho RJ, et al. Pharmacy refill adherence outperforms self-reported methods in predicting HIV therapy outcome in resource-limited settings. Bmc Public Health. 2014;14:11.

168. Law A, Lee YC, Gorritz M, Plouffe L. Does switching contraceptive from oral to a patch or vaginal ring change the likelihood of timely prescription refill? Contraception. 2014;90(2):188-94.

169. Vervloet M, van Dijk L, de Bakker DH, Souverein PC, Santen-Reestman J, van Vlijmen B, et al. Short- and long-term effects of real-time medication monitoring with short message service (SMS) reminders for missed doses on the refill adherence of people with Type 2 diabetes: evidence from a randomized controlled trial. Diabetic Medicine. 2014;31(7):821-8.

170. Wang JY, Lee SH, Lee IT, Chen JD, Sheu WHH. Effect of prescription refill on quality of care among patients with type 2 diabetes: An exploratory study. Diabetes Res Clin Pract. 2014;105(1):110-8.

171. Garth B, Temple-Smith M, Clark M, Hutton C, Deveny E, Biezen R, et al. 'Your lack of organisation doesn't constitute our emergency' - repeat prescription management in general practice. Aust Fam Physician. 2014;43(6):404-8.

172. Jonsson AK, Schioler L, Lesen E, Sundell KA, Mardby AC. Influence of refill adherence method when comparing level of adherence for different dosing regimens. Eur J Clin Pharmacol. 2014;70(5):589-97.

173. Sarkar U, Lyles CR, Parker MM, Allen J, Nguyen R, Moffet HH, et al. Use of the Refill Function Through an Online Patient Portal is Associated With Improved Adherence to Statins in an Integrated Health System. Med Care. 2014;52(3):194-201.

174. Weitoft GR, Berglund M, Lindstrom EA, Nilsson M, Salmi P, Rosen M. Mortality, attempted suicide, re-hospitalisation and prescription refill for clozapine and other antipsychotics in Sweden-a register-based study. Pharmacoepidemiol Drug Saf. 2014;23(3):290-8.

175. Petty DR, Zermansky AG, Alldred DP. The scale of repeat prescribing - time for an update. BMC Health Serv Res. 2014;14:4.

176. Chen LC, Chen TC, Huang YB, Chang CS. Disease acceptance and adherence to imatinib in Taiwanese chronic myeloid leukaemia outpatients. Int J Clin Phar,. 2014;36(1):120-7.

177. Lesen E, Sundell KA, Carlsten A, Mardby AC, Jonsson AK. Is the level of patient co-payment for medicines associated with refill adherence in Sweden? Eur J Public Health. 2014;24(1):85-90.

178. Nakiwogga-Muwanga A, Katabira E, Kiragga A, Kambugu A, Nakibuuka-Lubwama E, Manabe YC, et al. Factors before enrolment are associated with being removed from a Pharmacy-only Refill Programme at a large urban HIV/AIDS clinic, Uganda. Int J STD AIDS. 2014;25(2):105-12.

179. Blais L, Vilain A, Kettani FZ, Forget A, Lalonde G, Beauchesne MF, et al. Accuracy of the days' supply and the number of refills allowed recorded in Quebec prescription claims databases for inhaled corticosteroids. BMJ Open. 2014;4(11):8.

180. Van Steenis MNA, Driesenaar JA, Bensing JM, Van Hulten R, Souverein PC, Van Dijk L, et al. Relationship between medication beliefs, self-reported and refill adherence, and symptoms in patients with asthma using inhaled corticosteroids. Patient Prefer Adherence. 2014;8:83-91.

181. Vinikoor MJ, Schuttner L, Moyo C, Li M, Musonda P, Hachaambwa LM, et al. Short Communication: Late Refills During the First Year of Antiretroviral Therapy Predict Mortality and Program Failure Among HIV-Infected Adults in Urban Zambia. Aids Res Hum Retrovir. 2014;30(1):74-7.

182. Hedna K, Hagg S, Sundell KA, Petzold M, Hakkarainen KM. Refill adherence and self-reported adverse drug reactions and sub-therapeutic effects: a population-based study. Pharmacoepidemiol Drug Saf. 2013;22(12):1317-25.

183. Lichtner V, Venters W, Hibberd R, Cornford T, Barber N. The fungibility of time in claims of efficiency: The case of making transmission of prescriptions electronic in English general practice. Int J Med Inform. 2013;82(12):1152-70.

184. Neff MR, Aalsma MC, Rosenman MB, Wiehe SE. Psychiatric Medication Refill Practices of Juvenile Detainees. Child Psychiat Hum Dev. 2013;44(6):717-26.

185. Olsen KR, Hansen C, Abrahamsen B. Association between refill compliance to oral bisphosphonate treatment, incident fractures, and health care costs-an analysis using national health databases. Osteoporosis Int. 2013;24(10):2639-47.

186. Billups SJ, Delate T, Newlon C, Schwiesow S, Jahnke R, Nadrash A. Outcomes of a pharmacist-managed medication refill program. J Am Pharm Assoc. 2013;53(5):505-12.

187. Svarstad BL, Kotchen JM, Shireman TI, Brown RL, Crawford SY, Mount JK, et al. Improving refill adherence and hypertension control in black patients: Wisconsin TEAM trial. J Am Pharm Assoc. 2013;53(5):520-9.

188. Krousel-Wood M, Joyce C, Holt EW, Levitan EB, Dornelles A, Webber LS, et al. Development and Evaluation of a Self-Report Tool to Predict Low Pharmacy Refill Adherence in Elderly Patients with Uncontrolled Hypertension. Pharmacotherapy. 2013;33(8):798-811.

189. Hansen C, Pedersen BD, Konradsen H, Abrahamsen B. Anti-osteoporotic therapy in Denmark-predictors and demographics of poor refill compliance and poor persistence. Osteoporosis Int. 2013;24(7):2079-97.

190. Wilson PM, Kataria N, McNeilly E. Patient and carer experience of obtaining regular prescribed medication for chronic disease in the English National Health Service: a qualitative study. BMC Health Serv Res. 2013;13:11.

191. Andrade ASA, Deutsch R, Celano SA, Duarte NA, Marcotte TD, Umlauf A, et al. Relationships Among Neurocognitive Status, Medication Adherence Measured by Pharmacy Refill Records, and Virologic Suppression in HIV-Infected Persons. Jaids. 2013;62(3):282-92.

192. Ratanawongsa N, Karter AJ, Parker MM, Lyles CR, Heisler M, Moffet HH, et al. Communication and Medication Refill Adherence The Diabetes Study of Northern California. JAMA Intern Med. 2013;173(3):210-8.

193. Lin YF, Lin YM, Sheng LH, Chien HY, Chang TJ, Zheng CM, et al. First drive-through pharmacy services in Taiwan. J Chin Med Assoc. 2013;76(1):37-41.

194. Vaidya V, Gupte R, Balkrishnan R. Failure to refill essential prescription medications for asthma among pediatric Medicaid beneficiaries with persistent asthma. Patient Prefer Adherence. 2013;7:21-6.

195. Font R, Espinas JA, Gil-Gil M, Barnadas A, Ojeda B, Tusquets I, et al. Prescription refill, patient self-report and physician report in assessing adherence to oral endocrine therapy in early breast cancer patients: a retrospective cohort study in Catalonia, Spain. Br J Cancer. 2012;107(8):1249-56.

196. Glanz K, Beck AD, Bundy L, Primo S, Lynn MJ, Cleveland J, et al. Impact of a Health Communication Intervention to Improve Glaucoma Treatment Adherence. Arch Ophthalmol. 2012;130(10):1252-8.

197. Helmons PJ, Dalton AJ, Daniels CE. Effects of a direct refill program for automated dispensing cabinets on medication-refill errors. Am J Health-Syst Pharm. 2012;69(19):1659-64.

198. Cocohoba JM, Murphy P, Pietrandoni G, Guglielmo BJ. Improved antiretroviral refill adherence in HIV-focused community pharmacies. J Am Pharm Assoc. 2012;52(5):E67-E73.

199. Guirguis-Blake J, Keppel GA, Force RW, Cauffield J, Monger RM, Baldwin LM. Variation in Refill Protocols and Procedures in a Family Medicine Residency Network. Fam Med. 2012;44(8):564-8.

200. Clarke G, Dickerson J, Gullion CM, Debar LL. Trends in Youth Antidepressant Dispensing and Refill Limits, 2000 Through 2009. J Child Adolesc Psychopharmacol. 2012;22(1):11-20.

201. Molfenter TD, Bhattacharya A, Gustafson DH. The roles of past behavior and health beliefs in predicting medication adherence to a statin regimen. Patient Prefer Adherence. 2012;6:643-50.

202. Patel N, Sam A, Valentin A, Quan SF, Parthasarathy S. Refill Rates of Accessories for Positive Airway Pressure Therapy as a Surrogate Measure of Long-Term Adherence. J Clin Sleep Med. 2012;8(2):169-75.

203. Swinglehurst D, Greenhalgh T, Russell J, Myall M. Receptionist input to quality and safety in repeat prescribing in UK general practice: ethnographic case study. BMJ-British Medical Journal. 2011;343:11.

204. Ndubuka NO, Ehlers VJ. Adult patients' adherence to anti-retroviral treatment: A survey correlating pharmacy refill records and pill counts with immunological and virological indices. Int J Nurs Stud. 2011;48(11):1323-9.

205. Goel MS, Brown TL, Williams A, Hasnain-Wynia R, Thompson JA, Baker DW. Disparities in Enrollment and Use of an Electronic Patient Portal. J Gen Intern Med. 2011;26(10):1112-6.

206. Lesen E, Sandstrom TZ, Carlsten A, Jonsson AK, Mardby AC, Sundell KA. A comparison of two methods for estimating refill adherence to statins in Sweden: the RARE project. Pharmacoepidemiol Drug Saf. 2011;20(10):1073-9.

207. Gamble J, Stevenson M, Heaney LG. A study of a multi-level intervention to improve non-adherence in difficult to control asthma. Respir Med. 2011;105(9):1308-15.

208. Lee S, Rothbard AB, Noll E, Blank MB. Use of HIV and Psychotropic Medications among Persons with Serious Mental Illness and HIV/AIDS. Adm Policy Ment Health. 2011;38(5):335-44.

209. van den Boogaard J, Lyimo RA, Boeree MJ, Kibiki GS, Aarnoutse RE. Electronic monitoring of treatment adherence and validation of alternative adherence measures in tuberculosis patients: a pilot study. Bull World Health Organ. 2011;89(9):632-9.

210. Vinson MH, McCallum R, Thornlow DK, Champagne MT. Design, Implementation, and Evaluation of Population-Specific Telehealth Nursing Services. Nurs Econ. 2011;29(5):265-+.

211. Simons LA, Ortiz M, Calcino G. Persistence with a single pill versus two pills of amlodipine and atorvastatin: the Australian experience, 2006-2010. Med J Aust. 2011;195(3):134-7.

212. Farley JF, Wang CC, Hansen RA, Voils CI, Maciejewski ML. Continuity of Antipsychotic Medication Management for Medicaid Patients With Schizophrenia. Psychiatr Serv. 2011;62(7):747-52.

213. Valenstein M, Kavanagh J, Lee T, Reilly P, Dalack GW, Grabowski J, et al. Using A Pharmacy-Based Intervention To Improve Antipsychotic Adherence Among Patients With Serious Mental Illness. Schizophr Bull. 2011;37(4):727-36.

214. Wong MCS, Jiang JY, Griffiths SM. Adherence to lipid-lowering agents among 11,042 patients in clinical practice. Int J Clin Pract. 2011;65(7):741-8.

215. Simons LA, Ortiz M, Calcino G. Long term persistence with statin therapy Experience in Australia 2006-2010. Aust Fam Physician. 2011;40(5):319-22.

216. Zedler BK, Joyce A, Murrelle L, Kakad P, Harpe SE. A Pharmacoepidemiologic Analysis of the Impact of Calendar Packaging on Adherence to Self-Administered Medications for Long-Term Use. Clin Ther. 2011;33(5):581-97.

217. El-Khatib Z, Katzenstein D, Marrone G, Laher F, Mohapi L, Petzold M, et al. Adherence to Drug-Refill Is a Useful Early Warning Indicator of Virologic and Immunologic Failure among HIV Patients on First-Line ART in South Africa. Plos One. 2011;6(3):10.

218. Katic BJ, Rajagopalan S, Ho TW, Chen YT, Hu XH. Triptan persistency among newly initiated users in a pharmacy claims database. Cephalalgia. 2011;31(4):488-500.

219. de Boer IM, Prins JM, Sprangers MAG, Nieuwkerk PT. Using Different Calculations of Pharmacy Refill Adherence to Predict Virological Failure Among HIV-Infected Patients. Jaids. 2010;55(5):635-40.

220. Zima BT, Bussing R, Tang LQ, Zhang L, Ettner S, Belin TR, et al. Quality of Care for Childhood Attention-Deficit/Hyperactivity Disorder in a Managed Care Medicaid Program. J Am Acad Child Adolesc Psychiatr. 2010;49(12):1225-37.

221. Kripalani S, Gatti ME, Jacobson TA. Association of age, health literacy, and medication management strategies with cardiovascular medication adherence. Patient Education and Counseling. 2010;81(2):177-81.

222. Heisler M, Hofer TP, Klamerus ML, Schmittdiel J, Selby J, Hogan MM, et al. Study protocol: The Adherence and Intensification of Medications (AIM) study - a cluster randomized controlled effectiveness study. Trials. 2010;11:14.

223. Barron TI, Bennett K, Feely J. A Competing Risks Prescription Refill Model of Compliance and Persistence. Value Health. 2010;13(6):796-804.

224. Gagne JJ, Avorn J, Shrank WH, Schneeweiss S. Refilling and Switching of Antiepileptic Drugs and Seizure-Related Events. Clin Pharmacol Ther. 2010;88(3):347-53.

225. Patel NG, Lindsey T, Strunk RC, DeBaun MR. Prevalence of Daily Medication Adherence Among Children With Sickle Cell Disease: A 1-Year Retrospective Cohort Analysis. Pediatr Blood Cancer. 2010;55(3):554-6.

226. Liu YF, Yang M, Chao JD, Mulani PM. Greater refill adherence to adalimumab therapy for patients using specialty versus retail pharmacies. Adv Ther. 2010;27(8):523-32.

227. Goldman RE, Dube C, Lapane KL. Beyond the basics: Refills by electronic prescribing. Int J Med Inform. 2010;79(7):507-14.

228. Bailey JE, Wan JY, Tang J, Ghani MA, Cushman WC. Antihypertensive Medication Adherence, Ambulatory Visits, and Risk of Stroke and Death. J Gen Intern Med. 2010;25(6):495-503.

229. Ralston JD, Coleman K, Reid RJ, Handley MR, Larson EB. Patient Experience Should Be Part Of Meaningful-Use Criteria. Health Aff. 2010;29(4):607-13.

230. West JC, Wilk JE, Rae DS, Muszynski IL, Rubio-Stipec M, Alter CL, et al. First-Year Medicare Part D Prescription Drug Benefits: Medication Access and Continuity Among Dual Eligible Psychiatric Patients. J Clin Psychiatry. 2010;71(4):400-10.

231. Newby DA, Robertson J. Computerised prescribing: assessing the impact on prescription repeats and on generic substitution of some commonly used antibiotics. Med J Aust. 2010;192(4):192-5.

232. Lau R, Stewart K, McNamara KP, Jackson SL, Hughes JD, Peterson GM, et al. Evaluation of a community pharmacy-based intervention for improving patient adherence to antihypertensives: a randomised controlled trial. BMC Health Serv Res. 2010;10:7.

233. Duru OK, Schmittdiel JA, Dyer WT, Parker MM, Uratsu CS, Chan J, et al. Mail-Order Pharmacy Use and Adherence to Diabetes-Related Medications. Am J Manag Care. 2010;16(1):33-40.

234. Gazmararian J, Jacobson KL, Pan Y, Schmotzer B, Kripalani S. Effect of a Pharmacy-Based Health Literacy Intervention and Patient Characteristics on Medication Refill Adherence in an Urban Health System. Ann Pharmacother. 2010;44(1):80-7.

235. Hansen RA, Dusetzina SB, Dominik RC, Gaynes BN. Prescription refill records as a screening tool to identify antidepressant non-adherence. Pharmacoepidemiol Drug Saf. 2010;19(1):33-7.

236. Crockett J, Taylor S. Rural pharmacist perceptions of a project assessing their role in the management of depression. Aust J Rural Health. 2009;17(5):236-43.

237. Traveset EL, Mora RM, Sanz RT. Repeat prescription management systems: strategy for optimising their use. Aten Prim. 2009;41(9):487-92.

238. Saastamoinen LK, Klaukka TJ, Ilomaki J, Enlund H. An intervention to develop repeat prescribing in community pharmacy. J Clin Pharm Ther. 2009;34(3):261-5.

239. West JC, Wilk JE, Rae DS, Muszynski IS, Stipec MR, Alter CL, et al. Medicaid Prescription Drug Policies and Medication Access and Continuity: Findings From Ten States. Psychiatr Serv. 2009;60(5):601-10.

240. Hogue MD, Hogue HB, Lander RD, Avent K, Fleenor MA. The Nontraditional Role of Pharmacists After Hurricane Katrina: Process Description and Lessons Learned. Public Health Rep. 2009;124(2):217-23.

241. Vink NM, Klungel OH, Stolk RP, Denig P. Comparison of various measures for assessing medication refill adherence using prescription data. Pharmacoepidemiol Drug Saf. 2009;18(2):159-65.

242. Kripalani S, Risser J, Gatti ME, Jacobson TA. Development and Evaluation of the Adherence to Refills and Medications Scale (ARMS) among Low-Literacy Patients with Chronic Disease. Value Health. 2009;12(1):118-23.

243. Nietert PJ, Tilley BC, Zhao WL, Edwards PF, Wessell AM, Mauldin PD, et al. Two Pharmacy Interventions to Improve Refill Persistence for Chronic Disease Medications A Randomized, Controlled Trial. Med Care. 2009;47(1):32-40.

244. Corry M, McMurtry G. Implementing a repeat prescribing initiative in general practices within 12 months to ensure more robust and safer prescribing system for patients. Pharm World Sci. 2008;30(6):985-6.

245. Saastamoinen L, Enlund H, Klaukka T. Repeat prescribing in primary care: a prescription study. Pharm World Sci. 2008;30(5):605-9.

246. Law MR, Sournerai SB, Ross-Degnan D, Adams AS. A longitudinal study of medication nonadherence and hospitalization risk in schizophrenia. J Clin Psychiatry. 2008;69(1):47-53.

247. Krigsman K, Moen J, Nilsson JLG, Ring L. Refill adherence by the elderly for asthma/chronic obstructive pulmonary disease drugs dispensed over a 10-year period. J Clin Pharm Ther. 2007;32(6):603-11.

248. Krigsman K, Nilsson JLG, Ring L. Adherence to multiple drug therapies: refill adherence to concomitant use of diabetes and asthma/COPD medication. Pharmacoepidemiol Drug Saf. 2007;16(10):1120-8.

249. Lee PP, Walt JG, Chiang TH, Guckian A, Keener J. A gap analysis approach to assess patient persistence with glaucoma medication. Am J Ophthalmol. 2007;144(4):520-4.

250. Robin AL, Novack GD, Covert DW, Crockett RS, Marcic TS. Adherence in glaucoma: Objective measurements of once-daily and adjunctive medication use. Am J Ophthalmol. 2007;144(4):533-40.

251. Bryson CL, Au DH, Young B, McDonell MB, Fihn SD. A refill adherence algorithm for multiple short intervals to estimate refill compliance (ReComp). Med Care. 2007;45(6):497-504.

252. Krigsman K, Nilsson JLG, Ring L. Refill adherence for patients with asthma and COPD: comparison of a pharmacy record database with manually collected repeat prescriptions. Pharmacoepidemiol Drug Saf. 2007;16(4):441-8.

253. Krigsman K, Melander A, Carlsten A, Ekedahl A, Nilsson JLG. Refill non-adherence to repeat prescriptions leads to treatment gaps or to high extra costs. Pharm World Sci. 2007;29(1):19-24.

254. Seguy N, Diaz T, Campos DP, Veloso VG, Grinsztejin B, Teixeira L, et al. Evaluation of the consistency of refills for antiretroviral medications in two hospitals in the state of Rio de Janeiro, Brazil. Aids Care-Psychol Socio-Med Asp Aids-Hiv. 2007;19(5):617-25.

255. Gazmararian JA, Kripalani S, Miller MJ, Echt KV, Ren JL, Rask K. Factors associated with medication refill adherence in cardiovascular-related diseases: A focus on health literacy. J Gen Intern Med. 2006;21(12):1215-21.

256. Caetano PA, Lam JMC, Morgan SG. Toward a standard definition and measurement of persistence with drug therapy: Examples from research on statin and antihypertensive utilization. Clin Ther. 2006;28(9):1411-24.

257. Gardner EM, Burman WJ, Maravi ME, Davidson AJ. Durability of adherence to antiretroviral therapy on initial and subsequent regimens. Aids Patient Care STDS. 2006;20(9):628-36.

258. Hess LM, Raebel MA, Conner DA, Malone DC. Measurement of adherence in pharmacy administrative databases: A proposal for standard definitions and preferred measures. Ann Pharmacother. 2006;40(7-8):1280-8.

259. Nilsson JLG, Andersson K, Bergkvist A, Bjorkman I, Brismar A, Moen J. Refill adherence to repeat prescriptions of cancer drugs to ambulatory patients. Eur J Cancer Care. 2006;15(3):235-7.

260. Manfredi R, Sabbatani S, Orcioni GF, Martinelli GN, Chiodo F. Fatal long-term immunosuppressive therapy with uncontrolled repeat prescription. Ther Drug Monit. 2006;28(3):463-7.

261. Bautista-Arredondo S, Mane A, Bertozzi SA. Economic impact of antiretroviral therapy prescription decisions in the context of rapid scaling-up of access to treatment: lessons from Mexico. Aids. 2006;20(1):101-9.

262. Ferrell CW, Aspy CB, Mold JW. Management of prescription refills in primary care: An Oklahoma Physicians Resource/Research Network (OKPRN) study. J Am Board Fam Med. 2006;19(1):31-8.

263. Van Wijk BLG, Klungel OH, Heerdink ER, de Boer A. Refill persistence with chronic medication assessed from a pharmacy database was influenced by method of calculation. J Clin Epidemiol. 2006;59(1):11-7.

264. Andersson K, Melander A, Svensson C, Lind O, Nilsson JLG. Repeat prescriptions: refill adherence in relation to patient and prescriber characteristics, reimbursement level and type of medication. Eur J Public Health. 2005;15(6):621-6.

265. Miller AH, Larkin GL, Jimenez CH. Predictors of medication refill-seeking behavior in the ED. Am J Emerg Med. 2005;23(4):423-8.

266. Inciardi JE, Leeds AL. Assessing the utility of a community pharmacy refill record as a measure of adherence and viral load response in patients infected with human immunodeficiency virus. Pharmacotherapy. 2005;25(6):790-6.

267. Rijcken CAW, Tobi H, Vergouwen ACM, de Jong-van den Berg LTW. Refill rate of antipsychotic drugs: an easy and inexpensive method to monitor patients' compliance by using computerised pharmacy data. Pharmacoepidemiol Drug Saf. 2004;13(6):365-70.

268. Platt R, Reardon G, Mozaffari E. Observed time between prescription refills for newer ocular hypotensive agents: The effect of bottle size. Am J Ophthalmol. 2004;137:S17-S23.

269. Schectman JM, Schorling JB, Nadkarni MM, Voss JD. Can prescription refill feedback to physicians improve patient adherence? Am J Med Sci. 2004;327(1):19-24.

270. Buurma H, de Smet P, van den Hoff OP, Sysling H, Storimans M, Egberts ACG. Frequency, nature and determinants of pharmacy compounded medicines in Dutch community pharmacies. Pharm World Sci. 2003;25(6):280-7.

271. Rogers JE, Wroe CJ, Roberts A, Swallow A, Stables D, Cantrill JA, et al. Automated quality checks on repeat prescribing. Br J Gen Pract. 2003;53(496):838-44.

272. Delgado J, Heath KV, Yip B, Marion S, Alfonso V, Montaner JSG, et al. Highly active antiretroviral therapy: physician experience and enhanced adherence to prescription refill. Antivir Ther. 2003;8(5):471-8.

273. Newby DA, Fryer JL, Henry DA. Effect of computerised prescribing on use of antibiotics. Med J Aust. 2003;178(5):210-3.

274. Schectman JM, Bovbjerg VE, Voss JD. Predictors of medication-refill adherence in an indigent rural population. Med Care. 2002;40(12):1294-300.

275. Winkelaar PG. Lengthy prescription refills. Can Fam Phys. 2002;48:1447-.

276. Morningstar BA, Sketris IS, Kephart GC, Sclar DA. Variation in pharmacy prescription refill adherence measures by type of oral antihyperglycaemic drug therapy in seniors in Nova Scotia, Canada. J Clin Pharm Ther. 2002;27(3):213-20.

277. Zermansky AG, Petty DR, Raynor DK, Freemantle N, Vail A, Lowe CJ. Randomised controlled trial of clinical medication review by a pharmacist of elderly patients receiving repeat prescriptions in general practice. Br Med J. 2001;323(7325):1340-3.

278. Vanelli M, Burstein P, Cramer J. Refill patterns of atypical and conventional antipsychotic medications at a national retail pharmacy chain. Psychiatr Serv. 2001;52(9):1248-50.

279. Shapiro NL, Breen M, Mategrano VA. Patient satisfaction with a scheduled prescription-refill service. Am J Health-Syst Pharm. 2001;58(4):322-5.

280. Connolly JP, McGavock H. Repeat prescribing: Which diagnoses,which drugs? Pharmacoepidemiol Drug Saf. 2000;9(4):305-11.

281. Bond C, Matheson C, Williams S, Williams P, Donnan P. Repeat prescribing: a role for community pharmacists in controlling and monitoring repeat prescriptions. Br J Gen Pract. 2000;50(453):271-5.

282. Boutet R, Wilcock M, Mackenzie I. Survey on repeat prescribing for acid suppression drugs in primary care in Cornwall and the Isles of Scilly. Aliment Pharmacol Ther. 1999;13(6):813-7.

283. Cox S, Wilcock P, Young J. Improving the repeat prescribing process in a busy general practice. A study using continuous quality improvement methodology. Qual Health Care. 1999;8(2):119-25.

284. McGavock H, Wilson-Davis K, Connolly JP. Repeat prescribing management - a cause for concern? Br J Gen Pract. 1999;49(442):343-7.

285. Dowell J, Cruikshank J, Bain J, Staines H. Repeat dispensing by community pharmacists: advantages for patients and practitioners. Br J Gen Pract. 1998;48(437):1858-9.

286. Hartley D. Repeat prescription: The national curriculum for initial teacher training. Br J Educ Stud. 1998;46(1):68-83.

287. Steiner JF, Prochazka AV. The assessment of refill compliance using pharmacy records: Methods, validity, and applications. J Clin Epidemiol. 1997;50(1):105-16.

288. Harris CM, Dajda R. The scale of repeat prescribing. Br J Gen Pract. 1996;46(412):649-53.

289. Taylor RJ. Repeat prescribing - Still our Achilles' heel? Br J Gen Pract. 1996;46(412):640-1.

290. Zermansky AG. Who controls repeats? Br J Gen Pract. 1996;46(412):643-7.

291. deVries CS, vanDiepen NM, Tromp TFJ, deJongvandenBerg LTW. Auditing GPs' prescribing habits: Cardiovascular prescribing frequently continues medication initiated by specialists. Eur J Clin Pharmacol. 1996;50(5):349-52.

292. Goudie BM, McKenzie PE, Cipriano J, Griffin EM, Murray FE. Repeat prescribing of ulcer healing drugs in general practice - Prevalence and underlying diagnosis. Aliment Pharmacol Ther. 1996;10(2):147-50.

293. Britten N, Brant S, Cairns A, Hall WW, Jones I, Salisbury C, et al. CONTINUED PRESCRIBING OF INAPPROPRIATE DRUGS IN GENERAL-PRACTICE. J Clin Pharm Ther. 1995;20(4):199-205.

294. Dowell JS, Snadden D, Dunbar JA. CHANGING TO GENERIC FORMULARY - HOW ONE FUNDHOLDING PRACTICE REDUCED PRESCRIBING COSTS. Br Med J. 1995;310(6978):505-8.

295. Skaer TL, Sclar DA, Markowski DJ, Won JKH. EFFECT OF VALUE-ADDED UTILITIES ON PRESCRIPTION REFILL COMPLIANCE AND HEALTH-CARE EXPENDITURES FOR HYPERTENSION. J Hum Hypertens. 1993;7(5):515-8.

296. Skaer TL, Sclar DA, Markowski DJ, Won JKH. EFFECT OF VALUE-ADDED UTILITIES ON PRESCRIPTION REFILL COMPLIANCE AND MEDICAID HEALTH-CARE EXPENDITURES - A STUDY OF PATIENTS WITH NON-INSULIN-DEPENDENT DIABETES-MELLITUS. J Clin Pharm Ther. 1993;18(4):295-9.

297. Skaer TL, Sclar DA, Markowski DJ, Won JKH. EFFECT OF VALUE-ADDED UTILITIES IN PROMOTING PRESCRIPTION REFILL COMPLIANCE AMONG PATIENTS WITH HYPERTENSION. Curr Ther Res-Clin Exp. 1993;53(3):251-5.

298. Cram DL, Stebbins M, Eom HS, Ratto N, Sugiyama D. PEER-REVIEW AS A QUALITY ASSURANCE MECHANISM IN 3 PHARMACIST-RUN MEDICATION-REFILL CLINICS. Am J Hosp Pharm. 1992;49(11):2727-30.

299. Sclar DA, Chin A, Skaer TL, Okamoto MP, Nakahiro RK, Gill MA. EFFECT OF HEALTH-EDUCATION IN PROMOTING PRESCRIPTION REFILL COMPLIANCE AMONG PATIENTS WITH HYPERTENSION. Clin Ther. 1991;13(4):489-95.

300. Drury M. SAFE PRACTICE IN REPEAT PRESCRIBING. Practitioner. 1990;234(1482):127-8.

301. Steele K, Mills KA, Gilliland AEW, Irwin WG, Taggart A. REPEAT PRESCRIBING OF NONSTEROIDAL ANTIINFLAMMATORY DRUGS EXCLUDING ASPIRIN - HOW CAREFUL ARE WE. Br Med J. 1987;295(6604):962-4.

302. Grant GB, Gregory DA, Vanzwanenberg TD. REPEAT PRESCRIBING - A STUDY PRIOR TO THE IMPOSITION OF THE LIMITED LIST. Journal of the Royal College of General Practitioners. 1986;36(285):148-50.

303. Ascione FJ, Brown GH, Kirking DM. EVALUATION OF A MEDICATION REFILL REMINDER SYSTEM FOR A COMMUNITY PHARMACY. Patient Education and Counseling. 1985;7(2):157-65.

304. Aylett M. COMPUTERIZED REPEAT PRESCRIPTIONS - SIMPLE SYSTEM. Br Med J. 1985;290(6475):1115-6.

305. Roland MO, Zander LI, Evans M, Morris R, Savage RA. EVALUATION OF A COMPUTER-ASSISTED REPEAT PRESCRIBING PROGRAM IN A GENERAL-PRACTICE. Br Med J. 1985;291(6493):456-8.

306. Difford F. PRACTICE RESEARCH - COMPUTER-CONTROLLED REPEAT PRESCRIBING USED TO ANALYZE DRUG MANAGEMENT. Br Med J. 1984;289(6445):593-5.

307. Difford F. REDUCING PRESCRIBING COSTS THROUGH COMPUTER-CONTROLLED REPEAT PRESCRIBING. Journal of the Royal College of General Practitioners. 1984;34(269):658-60.

308. Jacob A, Anderson RA. A MOVE TO A HEALTH-CENTER - THE EFFECT ON HOME VISITING, REPEAT PRESCRIBING AND PATIENTS CHOICE OF TRANSPORT. Journal of the Royal College of General Practitioners. 1984;34(264):381-5.

309. Nicol F, Gebbie H. REPEAT PRESCRIBING IN THE ELDERLY - A CASE FOR AUDIT. Scott Med J. 1984;29(1):21-4.

310. Telling JP, Davies KR, Difford F, Fornear JE, Reading CA. DEVELOPING A PRACTICE FORMULARY AS A BY-PRODUCT OF COMPUTER-CONTROLLED REPEAT PRESCRIBING. Br Med J. 1984;288(6432):1730-2.

311. Tullio CJ. PATIENT ACCEPTANCE OF A SPECIAL REFILL WINDOW. Milit Med. 1984;149(10):572-5.

312. Williams RF, Shepherd MD, Jowdy AW. EFFECT OF A CALL-IN PRESCRIPTION REFILL SYSTEM ON WORKLOAD IN AN OUTPATIENT PHARMACY. Am J Hosp Pharm. 1983;40(11):1954-6.

313. Bober KF, Purohit AA. REFILLING PRESCRIPTIONS AND PHYSICIAN CONSENT. Contemporary Pharmacy Practice. 1982;5(2):80-4.

314. Schulz RM, Gagnon JP. PATIENT BEHAVIOR PATTERNS REGARDING PRESCRIPTION REFILLS. Contemporary Pharmacy Practice. 1982;5(3):150-5.

315. Barlow M. REPEAT PRESCRIPTION CARD - PINK CARD. S Afr Med J. 1981;59(8):276-.

316. Meldrum D. PRACTICE RESEARCH - SIMPLE COMPUTERIZED REPEAT PRESCRIPTION CONTROL-SYSTEM. Br Med J. 1981;282(6280):1933-7.

317. Tulloch AJ. REPEAT PRESCRIBING FOR ELDERLY PATIENTS. Br Med J. 1981;282(6277):1672-5.

318. Everett CB. REPEAT PRESCRIPTION CARDS. Practitioner. 1974;213(1277):707-11.

319. Balint M. REPEAT PRESCRIPTION PATIENTS - ARE THEY AN IDENTIFIABLE GROUP. Psychiatry in Medicine. 1970;1(1):3-14.
